# Berberine Exerts Neuroprotective Effects in Alzheimer’s Disease by Switching Microglia M1/M2 Polarization Through PI3K-AKT Signaling

**DOI:** 10.33549/physiolres.935410

**Published:** 2025-02-01

**Authors:** Yikui HU, Peng ZHANG, Xun WANG

**Affiliations:** 1 Department of Neurology, Wuhan Wuchang Hospital, Wuchang Hospital Affiliated to Wuhan University of Science and Technology, Wuhan, Hubei, China; 2 Department of Gastroenterology, Wuhan Wuchang Hospital, Wuchang Hospital Affiliated to Wuhan University of Science and Technology, Wuhan, Hubei, China

**Keywords:** Alzheimer’s Disease, Berberine, Microglia polarization, Neuroinflammation, PI3K-AKT signaling

## Abstract

Berberine (BBR), a small molecule protoberberine isoquinoline alkaloid, is easy to cross the blood-brain barrier and is a potential drug for neurodegenerative diseases. Here, we explored the role and molecular mechanism of BBR in Alzheimer’s disease (AD) progression. Weighted gene co-expression network analysis (WGCNA) was conducted to determine AD pathology-associated gene modules and differentially expressed genes (DEGs) were also identified. GO and KEGG analyses were performed for gene function and signaling pathway annotation. Cell counting kit-8 (CCK8) assay was applied to analyze cell viability. Immunofluorescence (IF) staining assay was conducted to measure the levels of polarization markers. The production of inflammatory cytokines was analyzed by enzyme-linked immunosorbent assay (ELISA). Reactive oxygen species (ROS) level and mitochondrial membrane potential (MMP) were detected using a ROS detection kit and a MMP Detection Kit (JC-1), respectively. AD pathology-associated DEGs were applied for GO function annotation and KEGG enrichment analysis, and the results uncovered that AD pathology was related to immune and inflammation. Lipopolysaccharide (LPS) exposure induced the M1 phenotype of microglia, and BBR suppressed LPS-induced M1 polarization and induced microglia toward M2 polarization. Through co-culture of microglia and neuronal cells, we found that BBR exerted a neuro-protective role by attenuating the injury of LPS-induced HMC3 on SH-SY5Y cells. Mechanically, BBR switched the M1/M2 phenotypes of microglia by activating PI3K-AKT signaling. In **summary**, BBR protected neuronal cells from activated microglia-mediated neuro-inflammation by switching the M1/M2 polarization in LPS-induced microglia *via* activating PI3K-AKT signaling.

## Introduction

Alzheimer’s disease (AD) is a common progressive neurodegenerative disease that is a serious social issue due to the aging population, imposing a major burden on health care system [1]. The deposition of amyloid β (Aβ) plaques in the brain is a pathological feature of AD, leading to neurotoxicity and cognitive impairment [2]. Although multiple therapeutic drugs approved by the FDA have been used in the treatment of cognitive manifestations of AD, such as acetylcholine esterase inhibitor, the benefits are limited [3]. At present, there is no agent that can halt the progression of AD. Moreover, many AD cases require long-term nursing home care, which bring a huge economic burden on family and society. Therefore, it is urgently needed to seek novel effective agents to alleviate the burdens of AD patients and the whole society.

AD pathology is closely associated with microglia-mediated neuroinflammation, which executes immune-regulatory role to maintain physiological function [4]. Accumulating documents have pointed out that the activation of microglia is heterogeneous, including classic pro-inflammatory M1 phenotype and anti-inflammatory M2 phenotype [5]. Microglia in M1 polarization are featured by the release of pro-inflammatory cytokines (IL-1β, and IL-6, TNF-α) and mediators (iNOS and COX2), while microglia in M2 phenotype show the production of anti-inflammatory cytokines (IL-10) and mediators (ARG1) [6]. Microglia in M1 phenotype exacerbate the neuronal damage and restrain cell repair, whereas microglia in M2 phenotype play neuro-protective role and induce cell recovery [7]. Hence, agents that inhibit the M1 polarization and induce microglia toward M2 phenotype are ideal for AD treatment. Nevertheless, few agents have been reported to switch M1/M2 phenotype of microglia.

Traditional Chinese medicines have been utilized in China and other countries to prevent and treat neurodegenerative diseases for more than 3000 years [8]. Recently, many evidences have shown that the effective component from herbs presents good efficacies in AD treatment with few side effects [9,10]. The biological efficacies of berberine (BBR) have been identified in multiple diseases, including cancer [11], atherosclerosis [12], cardiovascular diseases [13], and neurodegenerative diseases [14]. In AD, Chen *et al*. reported that BBR diminished cognitive decline in AD mouse model by reducing tau level *via* an autophagy-based pathway [15]. Ye *et al*. found that BBR played a neuro-protective role in AD by recovering cerebral blood flow and decreasing Aβ accumulation [16]. However, the molecular mechanism by which BBR improved the cognitive impairment in AD remains largely unknown.

In the present study, the role of BBR in lipopolysaccharide (LPS)-induced microglia polarization was assessed, and the associated molecular mechanism was explored. Our study intends to provide the basis for microglia-based therapeutic methods in AD.

## Materials and Methods

### Weighted gene co-expression network analysis (WGCNA)

The gene expression information of 12 AD patients, 10 healthy old controls, and 8 healthy young controls was downloaded from Gene Expression Omnibus (GEO) database (https://www.ncbi.nlm.nih.gov/geo/). WGCNA package of the R platform was utilized to identify AD pathology-related gene modules with similar expression pattern according to the following procedures: (1) The weighted coefficient β was determined to satisfy the law of scale-free networks. (2) The hierarchical clustering tree was obtained and gene modules from the hierarchical clustering tree were identified using the dynamic tree cut method. (3) The module eigengene (ME) of each gene module was calculated and the correlation between the MEs of gene modules and interested traits was analyzed by Pearson correlation analysis, and the correlation coefficient and P value of each gene module were analyzed.

### Screening for differentially expressed genes (DEGs)

The gene expression information of 12 AD patients and 10 healthy old controls downloaded from GEO database was analyzed for DEGs. The DEGs were obtained with adj-P<0.05 and |Log2FC|>1 as the screening conditions. The data were represented as a volcano map.

### GO function annotation and KEGG enrichment analysis

The overlapping genes between three gene modules (MElightcyan, MElightyellow, and MEgreen) of WGCNA and the DEGs were applied for GO function annotation and KEGG enrichment analysis. GO was conducted for annotation visualization (DAVID; https://david.ncifcrf.gov/). KEGG analysis was performed to identify the main pathways affected by the overlapping genes.

### Cell culture

Human microglia cell line HMC3 and human neuroblastoma cell line SH-SY5Y were purchased from Procell Biotechnology (Wuhan, China). Two cell lines were routinely maintained in Dulbecco’s modified Eagle medium/nutrient mixture F-12 (DMEM/F12; Procell Biotechnology) plus 10 % FBS (Sunnbio Biotechnology, Wuhan, China) at 37 °C with 5 % CO_2_.

### Drug treatment

LPS (Cat. No. L8880) and BBR (Cat. No. SB8130) were purchased from Solarbio life sciences (Beijing, China). For LPS or BBR treatment alone, HMC3 cells were induced by LPS (1 μg/ml) or BBR (1 μM, 2 μM, 4 μM, 8 μM, 16 μM) for 24 h. For LPS and BBR co-treatment, HMC3 cells were pre-treated with BBR (2 μM, 4 μM, 8 μM) for 3 h followed by co-treated with BBR and LPS (1 μg/ml) for 24 h.

Amyloid-β oligomer (Aβ1-42) was purchased from Solarbio life sciences (Cat. No. A5990). SH-SY5Y cells were exposed to 20 μM Aβ1-42 (dissolved in DMSO and stored in 2 mM aliquots at −20 °C) for 24 h to establish AD cell model. For the co-culture of HMC3 cells and SH-SY5Y cells, SH-SY5Y cells were exposed to 20 μM Aβ1-42 for 24 h and the culture medium was replaced by the culture supernatant of HMC3 cells.

### Cell counting kit-8 (CCK8) assay

Cell viability was evaluated by CCK8 assay. Cells were seeded onto 96-well plates. After drug treatment, CCK8 reagent (Beyotime, Shanghai, China) was pipetted to the wells to incubate with cells for 4 h at 37 °C. A microplate reader (BioTek Instruments, Winooski, VT, USA) was utilized to examine the absorbance at 450 nm.

### Immunofluorescence (IF) staining assay

After the indicated treatment, cells were immobilized with 4 % paraformaldehyde, permeabilized with 0.1 % Triton X-100, sealed with 2 % bovine serum albumin (BSA), followed by incubation with the primary antibodies of iNOS (Cat. No. 18985-1-AP; 1:500; Proteintech, Wuhan, China), COX2 (Cat. No. 66351-1-Ig; 1:800; Proteintech), and ARG1 (Cat. No. 16001-1-AP; 1:500; Proteintech) overnight at 4 °C. Then, the secondary fluorescent antibodies were incubated with cells for another 1 h at 37 °C. Cell nuclear staining was conducted using DAPI (Cat. No. G1012; Servicebio, Wuhan, China) in a dark room, and cell images were captured using a confocal microscope.

### Enzyme-linked immunosorbent assay (ELISA)

The production of pro-inflammatory cytokines (IL-1β, IL-6, TNF-α) and anti-inflammatory cytokine (IL-10) was analyzed by ELISA using their corresponding kits, including Human IL-1β ELISA Kit (Cat. No. EK101B; LiankeBio, Hangzhou, China), Human IL-6 ELISA Kit (Cat. No. EK106; LiankeBio), Human TNF-α ELISA Kit (Cat. No. EK282; LiankeBio), and Human IL-10 ELISA Kit (Cat. No. EK110; LiankeBio). ELISA was implemented according to manufacturer’s instruction.

### 5,5′,6,6′-Tetrachloro-1,1′,3,3′-tetraethylbenzimidazolo-carbo-cyanine iodide (JC-1) staining assay

The mitochondrial membrane potential (MMP) was analyzed by JC-1 staining assay using MMP Detection Kit (JC-1) (Cat. No. C2006; Beyotime). In healthy mitochondria with a normal MMP, JC-1 aggregated to form a polymer, which emitted red fluorescence. In un-healthy mitochondria, JC-1 formed monomers due to the decline or loss of MMP, which emitted green fluorescence. After staining with JC-1 for 20 min at 37 °C, cells were rinsed with 1× JC-1 staining washing buffer twice. The fluorescence signal was detected under a confocal microscope.

### Detection of intracellular reactive oxygen species (ROS) level

The level of intracellular ROS was assessed by detecting the fluorescence of DCFH-DA-A using a commercial ROS detection kit (Cat. No. S0033; Beyotime). After the indicated treatment, cells were rinsed three times with 1× PBS, and then incubated with 5 μM DCFH-DA-A for 45 min at 37 °C away from light. Then, cells were detached with trypsin. The fluorescence signal was observed under a confocal microscope.

### Western blot assay

Cells were harvested and total protein was extracted using the RIPA buffer (Cat. No. P0013B; Beyotime) plus protease inhibitors. The concentrations of protein samples were measured using a BCA protein assay kit (Cat. No. P0012; Beyotime). Equal amount of protein samples (25 μg) was separated by SDS-PAGE and then transferred onto the PVDF membranes (Millipore, Billerica, MA, USA). The non-specific proteins in PVDF membranes were blocked by incubating with 5 % non-fat milk for 1 h. The membranes were incubated with the diluted primary antibodies overnight at 4 °C, including Cleaved-Casp3 (Cat. No. 19677-1-AP; 1:5000; Proteintech), Bax (Cat. No. 50599-2-Ig; 1:8000; Proteintech), p-PI3K (Cat. No. 17366S; 1:3000; Cell Signaling Technology, Danvers, MA, USA), PI3K (Cat. No. 4257S; 1:8000; Cell Signaling Technology), AKT (Cat. No. 9272s; 1:8000; Cell Signaling Technology), p-AKT (Cat. No. 9271s; 1:3000; Cell Signaling Technology), and GAPDH (Cat. No. 10494-1-AP; 1:20000; Proteintech). The next day, the membranes were incubated with the HRP-conjugated secondary antibody for 1 h at room temperature. A commercial ECL kit (Beyotime) was utilized to visualize the protein bands, and ImageJ software (NIH, Bethesda, MD, USA) was utilized for protein quantification.

### Data analysis

All experiments were carried out in triplicates unless otherwise mentioned. The data were analyzed by GraphPad Prism 8.0 software (GraphPad Prism, La Jolla, CA, USA) and expressed as mean ± standard deviation (SD). The correlation between MEs of gene modules and interested traits was analyzed by Pearson correlation analysis. The differences were analyzed by one-way ANOVA followed by Dunnett’s test. *P*<0.05 was considered to indicate a statistically significant difference.

## Results

### AD pathology-associated gene modules and DEGs are identified by bioinformatics analysis

**Fig. 1:**
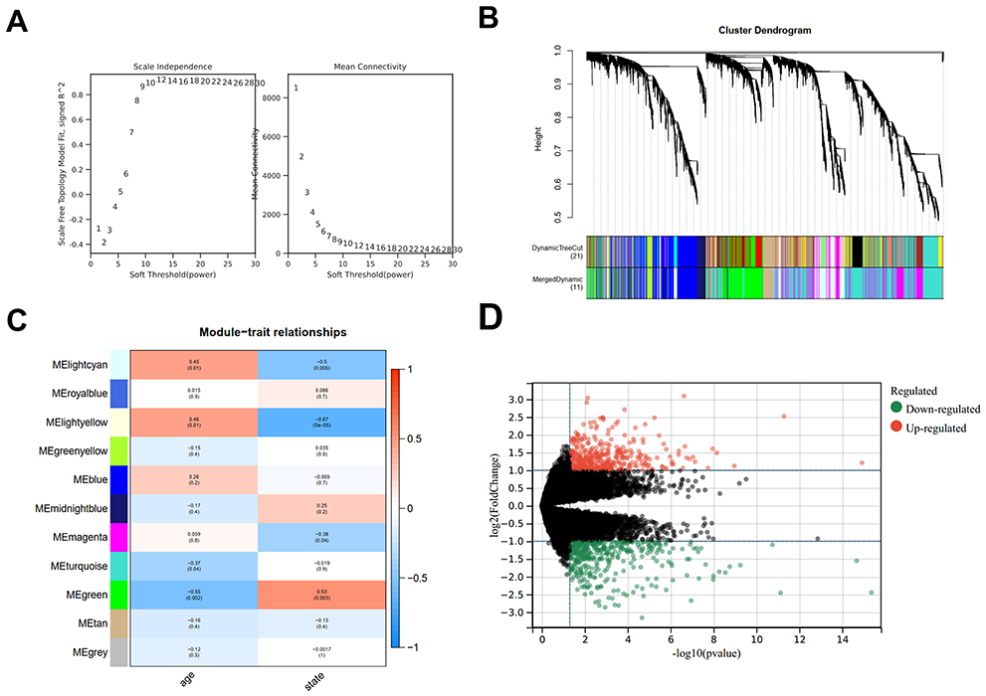
(**A**) Analysis of the weighted value β that satisfies the law of scale-free networks. (**B**) Each branch of the tree diagram represents genes, and a total of 11 gene modules were successfully clustered. (**C**) The correlation between the module eigengenes (MEs) and traits of interest (age and state) was assessed through a module-trait relationship analysis. Three gene modules (MElightcyan, MElightyellow, and MEgreen) clearly associated with age and state were selected for further analysis. (**D**) The differentially expressed genes (DEGs) in AD patients compared with old healthy samples were screened with the criteria of (adj-*P<*0.05 and |Log_2_FC|>1) and displayed as a volcano plot.

The gene expression information of 12 AD patients, 10 healthy old controls, and 8 healthy young controls was downloaded from GEO database and was applied for WGCNA. The soft threshold was determined using scale-free topology criterion. β=9 was picked to satisfy the scale-free network law (Fig. 1A). A total of 11 co-expression gene modules (MElightcyan, MEroyalblue, MElightyellow, MEgreenyellow, MEblue, MEmidnightblue, MEmagenta, MEturquoise, MEgreen, MEtan, MEgrey) were obtained (Fig. 1B). Then, the Pearson correlation coefficients between 11 gene modules and the clinical information (age and state) were analyzed to determine which modules were correlated with the clinical traits. The MElightcyan, MElightyellow, and MEgreen modules were chosen for further analysis due to their clear correlation with two clinical traits (Fig. 1C). The differentially expressed genes (DEGs) between AD patients and healthy old controls were identified *via* the gene differential expression analysis, including 213 up-regulated genes and 206 down-regulated genes (Fig. 1D). All DEGs are listed in Table S1.

### GO and KEGG annotation of AD progression-associated genes

**Fig. 2:**
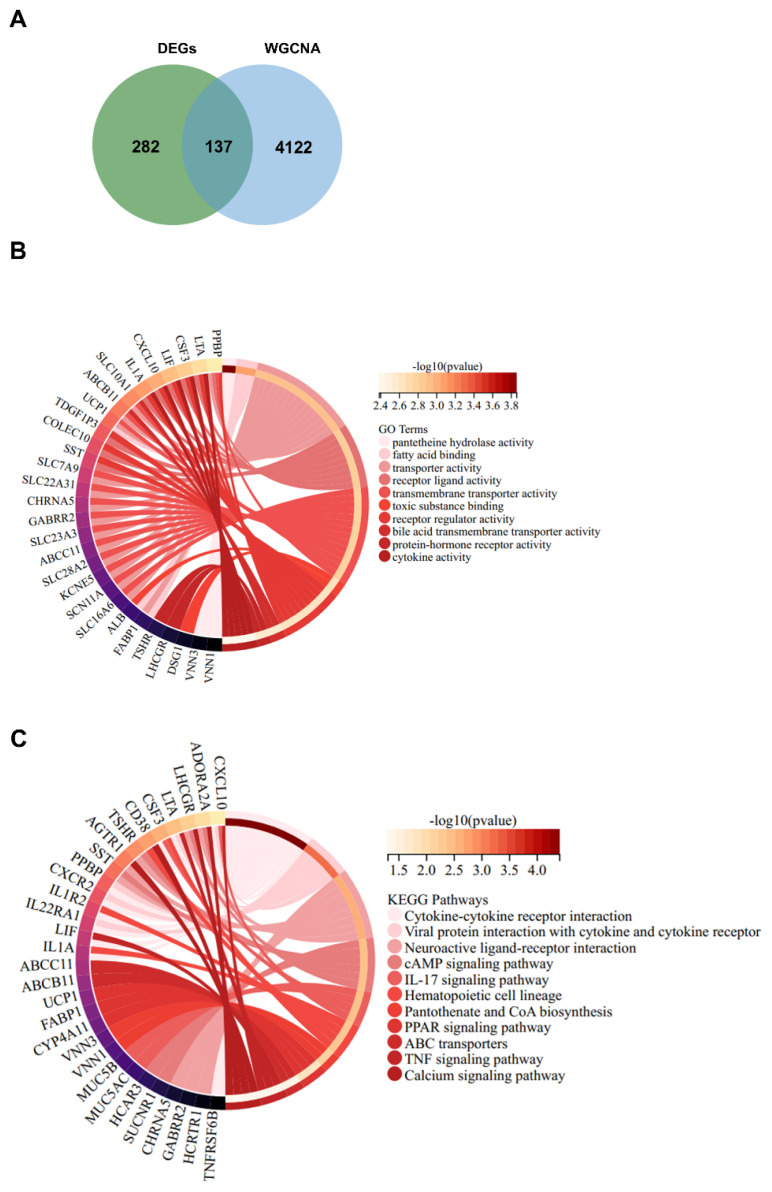
(**A**) Venn diagram was drawn to seek the overlapping genes between three gene modules (MElightcyan, MElightyellow, and MEgreen) of WGCNA and the differentially expressed genes in 12 AD patients and 10 healthy old controls. (**B** and **C**) The overlapping genes were subjected for functional and pathway annotation of GO and KEGG analyses.

DEGs and genes in three modules (MElightcyan, MElightyellow, and MEgreen) (listed in Table S2) of WGCNA were cross-linked to identify AD pathology-associated DEGs. As a result, a total of 137 overlapping genes were screened out (Fig. 2A). The overlapping genes were applied for GO (molecular function) and KEGG analyses. GO function annotation uncovered that these genes were associated with cytokine activity (Fig. 2B), which was involved in the regulation of neuroinflammation in AD [17]. KEGG enrichment analysis revealed that these genes were clearly related to several immune-associated pathways (Fig. 2C), including cytokine-cytokine receptor interaction [17], IL-17 signaling pathway [18], and TNF signaling pathway [19]. These data suggested that AD pathology was associated with immune and inflammation.

### BBR inhibits microglia M1 polarization in HMC3 cells

**Fig. 3:**
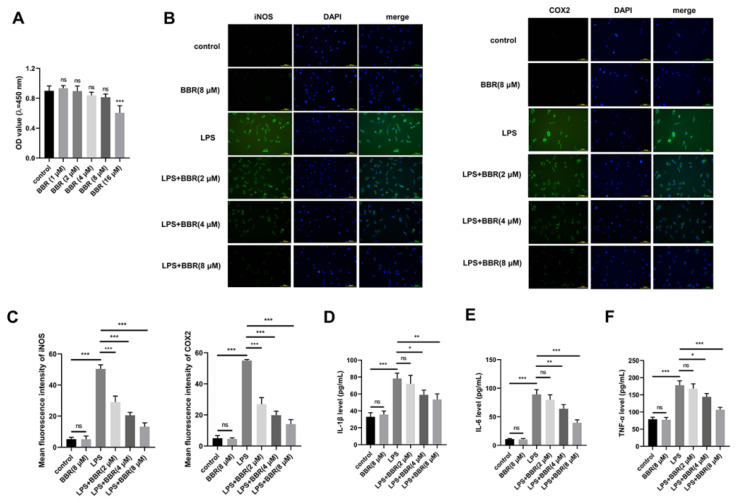
(**A**) CCK8 assay was conducted to analyze the toxicity of different doses of BBR (1 μM, 2 μM, 4 μM, 8 μM, or 16 μM) in HMC3 cells. (**B-F**) HMC3 cells were divided into six groups: control, BBR (8 μM), LPS, LPS+BBR (2 μM), LPS+BBR (4 μM), and LPS+BBR (8 μM). For BBR and LPS co-treated group, HMC3 cells were treated with BBR for 3 h, followed by LPS exposure for 24 h. (**B** and **C**) IF assay was carried out to evaluate the levels of two indicators of inflammation, including iNOS and COX2. (**D-F**) ELISA was implemented to analyze the release of three pro-inflammatory cytokines (IL-1β, IL-6, and TNF-α) in the culture supernatant. ns: no statistically significant difference, * *P<*0.05, ** *P<*0.01, *** *P<*0.001.

The neuroinflammation responses in AD pathology are mainly mediated by the resident brain immune microglia [20]. The classic M1 polarization of microglia causes the excessive production of pro-inflammatory mediators and neuro-toxic cytokines, leading to neuronal dysfunction or cell death [21]. Here, we exposed microglia HMC3 cells to LPS to induce cell activation and M1 polarization to mimic the pathological state of AD *in vivo*. To analyze the role of BBR in LPS-induced HMC3 cells, we first evaluated the effective doses of BBR. CCK8 assay was conducted to analyze the effect of BBR, from 1 μM to 16 μM, on the viability of HMC3 cells. The data revealed that BBR at the dose lower than 8 μM had no significant cytotoxicity on HMC3 cells (Fig. 3A). Therefore, BBR at the dose of 2 μM, 4 μM, or 8 μM was chosen in the following experiments. Subsequently, we assessed the biological role of BBR in LPS-induced HMC3 cells. iNOS and COX2 have been reported as two marker proteins in M1 microglia, while ARG1 is a marker protein in M2 microglia [22]. IF data showed that BBR alone had little effect on HMC3 polarization in the resting condition, and LPS alone induced M1 polarization of HMC3 cells, as evidenced by the significant up-regulation of iNOS and COX2 (Fig. 3B, C). Furthermore, the addition of BBR largely reversed LPS-induced up-regulation of iNOS and COX2 in a dose-dependent manner (Fig. 3B, C). ELISA showed that LPS induced the release of pro-inflammatory cytokines (IL-1β, IL-6, and TNF-α) in the culture supernatant, which were largely overturned by the addition of BBR (Fig. 3D-F), further indicating that BBR inhibited LPS-induced M1 polarization of HMC3 cells.

### BBR induces microglia toward M2 polarization in HMC3 cells

**Fig. 4:**
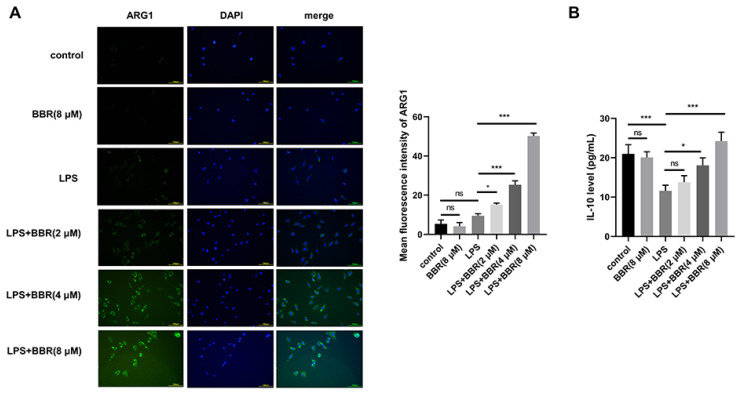
(**A**) The level of M2 polarization marker (ARG1) was measured by IF assay. (**B**) The concentration of anti-inflammatory factor IL-10 in the supernatant was detected by ELISA. ns: no statistically significant difference, * *P<*0.05, *** *P<*0.001.

We tested the level of M2 marker ARG1 and the release of anti-inflammatory cytokine IL-10 by IF and ELISA. BBR alone conferred little effect on HMC3 pola-rization in the resting condition, while BBR markedly increased the level of ARG1 and the promoted the release of IL-10 in LPS-induced HMC3 cells (Fig. 4A, B). These results demonstrated that BBR restrained LPS-induced M1 polarization and induced microglia toward M2 polarization.

### BBR treatment attenuates the injury of LPS-activated HMC3 microglia cells on Aβ1-42-induced AD cell model

**Fig. 5:**
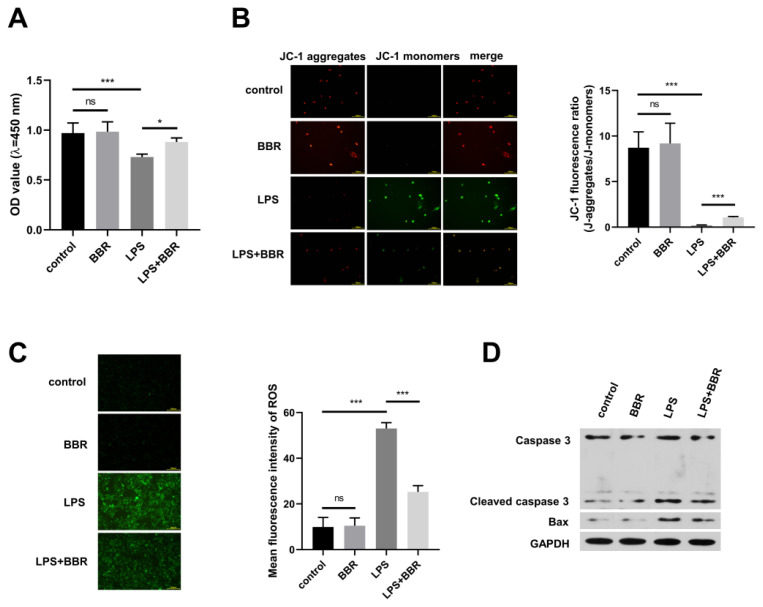
(**A-D**) Aβ_1-42_-exposed SH-SY5Y cells were cultured in the conditioned medium from control, BBR-stimulated, LPS-stimulated, or BBR and LPS co-treated HMC3 cells. (**A**) CCK8 assay was employed to analyze the viability of SH-SY5Y cells. (**B** and **C**) The MMP and ROS in SH-SY5Y cells were determined through IF staining method of JC-1 and DCFH-DA, respectively. (**D**) Western blot assay was conducted to measure the protein levels of two apoptosis-associated markers (cleaved CASP3 and Bax) in SH-SY5Y cells. ns: no statistically significant difference, * *P<*0.05, *** *P<*0.001.

AD is a common neurodegenerative disease featured by the accumulation of Aβ in the brain. Here, SH-SY5Y cells were exposed to Aβ1-42 for 24 h to establish AD cell model, and then we applied the conditioned medium from BBR or LPS-induced HMC3 cells to AD cell model. Compared with untreated HMC3 cells-derived conditioned medium, BBR-induced HMC3 cells-derived conditioned medium had almost no effect on the viability of SH-SY5Y cells, and LPS-exposed HMC3 cells-derived conditioned medium significantly reduced the viability of SH-SY5Y cells (Fig. 5A). In addition, the administration of BBR prominently relieved the cytotoxic effect of activated HMC3 cells on SH-SY5Y cells (Fig. 5A). The oxidative damage of activated HMC3 cells on SH-SY5Y cells was evaluated by measuring mitochondrial membrane potential (MMP) and ROS level. Activated HMC3 cells mediated by LPS induced JC-1 aggregates (red) to JC-1 monomers (green) in SH-SY5Y cells (Fig. 5B), suggesting that activated HMC3 cells induced the dysfunction of mitochondria in SH-SY5Y. Meanwhile, activated HMC3 cells mediated by LPS markedly elevated the level of ROS (Fig. 5C). In addition, the addition of BBR significantly attenuated the oxidative injury of activated HMC3 cells on SH-SY5Y (Fig. 5B, C). Western blot assay was employed to analyze the apoptosis of SH-SY5Y cells. The protein levels of Cleaved-Casp3 and Bax in SH-SY5Y cells were up-regulated when cultured with LPS-induced HMC3 cells-derived medium (Fig. 5D), suggesting that activated HMC3 cells induced SH-SY5Y cell apoptosis. However, this effect was partly reversed by the addition of BBR (Fig. 5D). In conclusion, BBR protected SH-SY5Y cells from activated HMC3 cells-induced injury.

### BBR promotes microglia M2 polarization through PI3K-AKT pathway

**Fig. 6:**
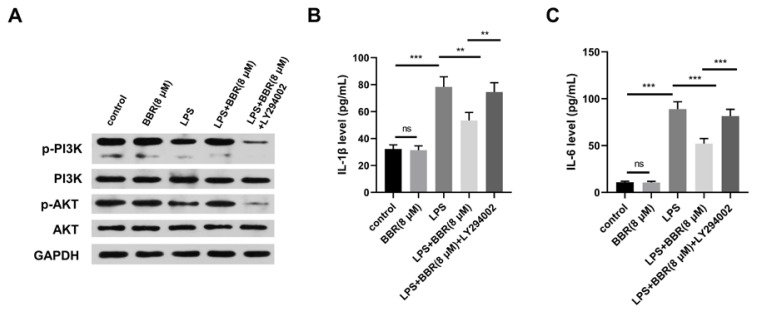
(**A-C**) HMC3 cells were divided into five groups: control, BBR (8 μM), LPS, LPS+BBR (8 μM), LPS+BBR (8 μM) + LY294002. For BBR and LPS co-treated group, HMC3 cells were treated with BBR for 3 h, followed by LPS exposure for 24 h. For LPS, BBR, and LY294002 co-treated group, HMC3 cells were treated with BBR for 3 h, followed by LPS and LY294002 stimulation for 24 h. (**A**) The levels of p-PI3K, PI3K, p-AKT, and AKT in HMC3 cells were determined by western blot assay. (**B** and **C**) ELISA was employed to detect the release of two pro-inflammatory cytokines (IL-1β and IL-6) in the culture supernatant of HMC3 cells. ns: no statistically significant difference, ** *P<*0.01, *** *P<*0.001.

To investigate the mechanism by which BBR affected the polarization of microglia HMC3 cells, we detected the effect of BBR on the activity of PI3K-AKT pathway, which is a well-known AD pathology-related pathway [23]. We found that BBR alone had little regulatory effects on the phosphorylation levels of PI3K and AKT, while LPS alone markedly down-regulated the phosphorylation levels of PI3K and AKT (Fig. 6A), indicating that LPS exposure inactivated PI3K-AKT signal pathway in HMC3 cells. In addition, the co-treatment of BBR and LPS partly rescued the activity of PI3K-AKT signaling, and this effect was reversed again by the addition of PI3K-AKT pathway inhibitor LY294002 (Fig. 6A). LPS induced the release of two pro-inflammatory cytokines in HMC3 cells, including IL-1β and IL-6, which were overturned by the administration of BBR (Fig. 6B, C). Moreover, the addition of LY294002 markedly reversed the effect of BBR on the production of IL-1β and IL-6 (Fig. 6B, C), suggesting that BBR regulated the inflammation of HMC3 cells through PI3K-AKT pathway. In a word, BBR induced HMC3 cells toward M2 polarization through PI3K-AKT pathway.

## Discussion

AD is a common neurodegenerative disorder and is also the most common cause of dementia. At present, the etiology and pathogenesis of AD remain unclear. Here, we obtained gene expression information of AD patients, healthy old controls, and healthy young controls from GEO database and identified three AD pathology-associated gene modules by WGCNA. Meanwhile, DEGs between AD patients and healthy old controls were analyzed. Then, the overlapping genes between DEGs and three genes modules were screened out as AD-associated dysregulated genes. Through GO and KEGG analyses, these genes were found to be correlated with inflammation-related pathways, indicating that inflammation-related pathways were involved in AD pathogenesis.

Microglia-mediated neuroinflammation is a pivotal factor in AD pathogenesis. Microglia can be activated through a classic or an alternative pathway, named M1 or M2 polarization, respectively. M1 or M2 microglia exhibit diverse properties and roles, which are differentially implicated in AD pathogenesis. Microglia with M1 polarization promote the process of inflammation by increasing the release of pro-inflammatory cytokines and mediators, which can be activated by LPS or IFN-γ and contribute to the inflammatory responses especially in neurodegenerative diseases [24]. M2 microglia exhibit anti-inflammatory phenotype by up-regulating the levels of anti-inflammatory cytokines and mediators, which can be activated by IL-4 and contribute to tissue repair and neuroprotection [24]. In addition, M1 and M2 phenotypes can be mutually transformed under multiple conditions. For instance, Mohanraj *et al*. reported that trehalose-6,6′-dibehenate could down-regulate the markers (IL-1β and IL-6) of M1 polarization induced by LPS and increased the levels of M2 markers (ARG1 and YM1/2) [25]. Furthermore, Han *et al*. pointed out that TOPK, a mitogen-activated protein kinase, contributed to M2 polarization of microglia possibly by reducing the activities of histone deacetylases [26]. These documents suggested that M1/M2 polarization of microglia could be modulated through multiple pathways.

Recently, traditional Chinese medicine extracted from herbs are reported to have good efficacies to AD. For example, EGB761, an effective constitute of Ginkgo biloba, is reported to improve the cognitive function and neuropsychiatric symptoms in AD [9]. BBR is a protoberberine isoquinoline alkaloid with various biological activities, such as anti-microbial, anti-tumor, and immuno-regulatory activities [27]. BBR was found to be a small molecule with a molecular weight of only 371.8 Da and was easy to cross the blood-brain barrier [28]. Previous pharmacokinetic investigations of BBR in rat models have demonstrated that BBR swiftly traverses the blood-brain barrier within 0.2 h post-administration, reaching peak concentration in the brain between 2 to 4 h, followed by a gradual elimination phase [29,30]. Currently, the molecular mechanism of BBR penetrating the blood-brain barrier has not been fully elucidated. A previous study reported that BBR could influence the permeability of the blood-brain barrier by enhancing the level of the integral membrane protein, claudin-5 [31]. Previous articles suggested that BBR might remit AD progression by regulating amyloid plaques in extracellular space and neurofibrillary tangles in intracellular space [32]. In this study, we explored the role of BBR on M1/M2 polarization of microglia. We found that LPS-induced M1 polarization of microglia could be overturned by BBR, suggesting that BBR exerted a neuroprotective effect on LPS-induced microglia by switching M1/M2 polarization. Then, we explored whether BBR protected neuron cells from injury mediated by LPS-induced M1 microglia. Through establishing co-culture model of microglia and neuronal cells, we found that BBR attenuated the injury of LPS-activated HMC3 on Aβ-induced AD cell model.

Subsequently, the working mechanism by which BBR switched microglia M1/M2 polarization was explored. BBR has been reported to suppress carotid atherosclerosis in high-fat diet-established ApoE^−/−^ mice by regulating cell autophagy, proliferation, and apoptosis *via* PI3K-AKT-mTOR signaling [33]. Tong *et al*. reported that BBR alleviated gastric tissue injury in chronic atrophic gastritis rats possibly through regulating TGF-β1-PI3K-AKT pathway [34]. Wang *et al*. pointed out that BBR exerted anti-inflammatory and anti-apoptotic efficacies in myocardial cells under ischemia-reperfusion through NF-κB and PI3K/AKT pathways [35]. These data indicated that PI3K-AKT signaling was an important downstream pathway by which BBR exerted biological properties. Accumulating evidence have suggested that PI3K-AKT signaling exerted a vital role in the central nervous system, including the proliferation and differentiation of neuronal cells and neurogenesis [36,37]. In AD, activated PI3K-AKT delayed AD progression through multiple ways, including the regulation of inflammatory and immune responses [23]. Here, we measured the activity of PI3K-AKT signaling in microglia with LPS treatment alone or together with BBR. LPS exposure markedly suppressed the activity of PI3K-AKT signaling, leading to the release of pro-inflammatory cytokines. And these effects were largely overturned by the addition of BBR. These results demonstrated that BBR regulated HMC3 cells toward M2 polarization through PI3K-AKT pathway.

The impairment of the clearance of metabolic wastes from the brain *via* the glymphatic system has been reported to be involved in the progression of several neurodegenerative diseases such as AD [38,39]. This is a strong research trend that aims to physically remove metabolic waste, in the opposite direction of targeted therapies that look for underlying molecules. In our study, we explored pharmacological interventions to regulate microglia function and inflammatory response, thereby reducing the production of metabolic wastes. Although the two lines of treatment approaches from different directions, both are moving toward the goal of alleviating or slowing the progression of AD. The two are potentially complementary, and a combination of the two may provide a more comprehensive therapeutic effect for AD in the future.

In conclusion, our data showed that BBR promoted the activation of PI3K-AKT signaling, decreased the M1 polarization of microglia and promoted microglia polarization toward M2 phenotype, thus alleviating the neuroinflammation and reducing the injury of neuronal cells. These evidence indicated that BBR might have substantial value in the treatment of neuroinflammation-related diseases, including AD.

## Supplementary Information
