# Supplementary material for: Berberine Exerts Neuroprotective Effects in Alzheimer’s Disease by Switching Microglia M1/M2 Polarization Through PI3K-AKT Signaling
Source: Physiol Res. 2025 Feb 1;74(1):129–40. doi: 10.33549/physiolres.935410 (PMC11995938; doi:10.33549/physiolres.935410)
Supplement: Supplementary file 1 [file 74_129_TableS1.pdf]

Tag	logFC	pvalue	FDR
LOC105376772	3,192319	5,52E-05	0,003456
VTRNA2-1	3,092399	2,35E-07	8,48E-05
LRIT1	2,709757	0,00014	0,006182
SGO1	2,521375	5,16E-12	2,19E-08
UNQ6494	2,486912	0,001449	0,026556
OR7A5	2,481034	5,62E-06	0,000771
MIR6847	2,47614	0,001405	0,025971
NAA11	2,470489	0,00189	0,031238
NLRP4	2,421869	0,001342	0,025147
NANOS2	2,375298	0,00029	0,009605
MIR6876	2,35936	0,003647	0,045739
LOC101927870	2,310503	0,000352	0,010935
SERPINA5	2,297291	1,54E-05	0,001496
LINC01193	2,255306	0,002768	0,039286
LOC100507616	2,213811	0,000516	0,014171
FSCN3	2,196178	4,38E-05	0,002979
CEACAM16	2,182529	0,002912	0,040381
OIT3	2,137146	0,000773	0,018014
LINC01068	2,022562	0,000828	0,01865
LOC102724484	1,988044	0,000138	0,006131
LINC01546	1,979692	3,42E-06	0,000564
CHRM5	1,951602	0,003529	0,044958
FOXJ1	1,884803	5,41E-05	0,003422
LINC00463	1,87756	1,94E-05	0,001733
CTRB1	1,870673	0,000583	0,015336
ST8SIA6-AS1	1,863748	0,003095	0,041877
IHH	1,811572	0,001783	0,030125
ZNF341-AS1	1,810826	5,16E-05	0,003307
ADORA2A	1,784277	1,89E-05	0,001718
CYP4B1	1,777853	8,08E-05	0,004343
LINC01436	1,744618	0,001273	0,02424
LOC100506136	1,744559	2,11E-06	0,000416
LOC100288866	1,743608	0,000864	0,019135
LINC00906	1,732171	0,002054	0,032876
KCTD14	1,715408	0,001776	0,030125
LOC101929151	1,705933	0,000707	0,017146
C7orf61	1,671454	2,15E-07	7,89E-05
CLEC18B	1,660744	0,000335	0,01056
GMNC	1,646552	0,000161	0,006676
IP6K3	1,628866	0,003002	0,041203
LOC102724596	1,607136	1,09E-08	1,16E-05
KIF19	1,603864	0,000233	0,008292
LTA	1,599544	0,000843	0,018824
PROK2	1,597557	0,00143	0,026308
FAAHP1	1,591741	1,58E-06	0,00034

SLC23A3	1,572411	0,000247	0,00867
UCKL1-AS1	1,562894	7,27E-05	0,004108
SCNN1G	1,558814	0,000821	0,01865
HIST2H2BC	1,545995	0,000303	0,009802
LBX2	1,544291	4,14E-07	0,000126
LOC102725079	1,543427	0,000215	0,007972
SALRNA2	1,53811	0,001783	0,030125
PRR34	1,536861	0,000113	0,005403
LINC01338	1,536569	3,00E-05	0,002325
LOC284933	1,534821	0,000299	0,009779
LOC108783645	1,527252	0,000137	0,006099
CXCR1	1,515925	0,000225	0,008143
MIR5690	1,513194	0,002935	0,040538
MIR194-2HG	1,501881	0,003031	0,04144
GNRH1	1,493781	6,97E-09	9,27E-06
LOC149684	1,490946	6,42E-05	0,003793
VTRNA1-1	1,483403	0,000173	0,006966
OPTC	1,480469	0,000791	0,018283
EPS8L3	1,480285	0,00026	0,008946
HIGD1C	1,473868	0,000311	0,010021
POU2AF1	1,471761	0,002695	0,038781
OLAH	1,470958	0,000895	0,019685
LINC00323	1,459247	0,001555	0,027945
LINC01134	1,450422	0,001123	0,022582
TSPAN10	1,442797	0,001535	0,027692
GABRR2	1,440733	4,23E-07	0,000127
MIR6717	1,440434	2,47E-05	0,002014
LINC00514	1,438905	0,002749	0,039187
LOC727993	1,435692	0,00181	0,030496
CXCR2	1,431537	0,001164	0,023018
STON1	1,428396	0,000199	0,007558
LOC100129316	1,422502	2,55E-05	0,002057
DNAJB5-AS1	1,42097	0,003987	0,048
CHRNA5	1,41122	1,55E-05	0,001496
LOC100507468	1,404455	0,003971	0,047902
THRSP	1,399498	0,000346	0,010819
AZGP1	1,398911	7,20E-05	0,004103
MIR6758	1,395101	0,002661	0,038501
FAM222A-AS1	1,394001	0,000177	0,00707
EN2	1,393282	0,002273	0,034848
PRR34-AS1	1,384214	1,05E-06	0,000251
OVOL3	1,377302	3,47E-05	0,00256
LINC01561	1,374742	0,000108	0,005224
NEAT1	1,373374	8,89E-08	4,40E-05
LBX2-AS1	1,373213	0,000145	0,006326
RASA4	1,369911	0,001812	0,030496

SLC25A30-AS1	1,360759	0,00035	0,010911
MRGPRF	1,360409	1,25E-05	0,001295
NMRK2	1,354452	0,002005	0,032384
LOC101928269	1,354331	0,000793	0,018305
HPN-AS1	1,353388	0,002188	0,034074
ABCC2	1,352406	4,23E-06	0,000651
ELMO1-AS1	1,352115	0,000305	0,009815
SH2D6	1,35157	1,80E-07	6,72E-05
TNFRSF6B	1,340321	0,000188	0,007321
ADAMTS2	1,33947	2,96E-05	0,002306
ALOX15B	1,334523	0,000158	0,006607
RNU11	1,334329	0,002867	0,040017
TNRC6C-AS1	1,332137	0,000109	0,005243
SLC45A3	1,330041	0,003407	0,044033
LINC00638	1,320541	1,30E-07	5,75E-05
TFCP2L1	1,309849	9,34E-06	0,001085
ADRA2B	1,308393	1,44E-05	0,001459
LOC101929054	1,29416	2,39E-05	0,001977
LDLRAP1	1,280606	0,000723	0,017358
SNORD22	1,276218	0,000831	0,01865
ADORA2A-AS1	1,274148	0,00174	0,029827
LINC00304	1,267775	0,002387	0,035957
RRAD	1,254815	0,001163	0,023013
GTSE1-AS1	1,248336	8,73E-07	0,00022
GPR141	1,245681	0,00032	0,010225
PCP2	1,243231	0,001747	0,029867
FAM53B-AS1	1,240901	7,35E-08	3,91E-05
LINC00926	1,233035	0,000506	0,014001
SERTAD4-AS1	1,228129	0,000259	0,008934
HEATR9	1,226959	0,000917	0,019961
CLDN9	1,226568	4,05E-05	0,002841
UHRF1	1,221582	6,38E-05	0,003779
ATAD3C	1,221406	1,54E-05	0,001496
SLC10A1	1,219642	0,00045	0,012989
STAG3L1	1,218802	0,002687	0,038747
MIR1914	1,218304	0,0008	0,018407
KLF15	1,210084	1,20E-15	1,27E-11
ARRDC2	1,204402	0,000895	0,019685
LMNTD1	1,200426	0,000226	0,008158
LOC101927043	1,195366	0,000146	0,006342
SLC35G6	1,188751	0,000921	0,020038
SNORA54	1,187793	0,001148	0,022856
LINC00601	1,187024	0,003618	0,04557
SLC4A9	1,184424	2,25E-05	0,001907
GNAS-AS1	1,180202	0,001225	0,023781
SAMD11	1,173246	0,000559	0,014798

GJA9-MYCBP	1,17216	7,33E-07	0,000198
SPACA6P-AS	1,170842	8,79E-07	0,00022
C20orf173	1,170656	0,000471	0,013319
ITPKB-IT1	1,170509	1,47E-05	0,00147
LMNTD2	1,166234	0,003975	0,047902
C6orf118	1,162896	0,000149	0,006371
SLC5A3	1,158893	8,18E-05	0,004382
FANCB	1,153674	0,00175	0,029896
AKR1C2	1,150198	9,94E-06	0,001137
OVCH2	1,143388	0,000622	0,015791
WDR38	1,136736	4,67E-05	0,003097
SHISA2	1,1364	0,001248	0,024002
SAP25	1,134884	1,79E-05	0,001667
FOXD2-AS1	1,133235	0,001314	0,024772
HIF3A	1,132719	8,44E-05	0,004451
ERN2	1,132166	0,001473	0,026864
CCNA2	1,13011	1,21E-05	0,001259
LDHAL6B	1,129364	0,001041	0,021595
LINC01088	1,127575	0,002834	0,039812
SNORA55	1,126064	0,000204	0,007697
LRRC46	1,12564	0,000644	0,016088
PRKX	1,123173	1,07E-09	2,06E-06
QDPR	1,121329	0,000387	0,011626
HSPB7	1,112689	3,80E-05	0,002701
PMEL	1,112251	0,001639	0,02874
DNAH11	1,110603	2,40E-06	0,00045
CEL	1,105722	0,002615	0,03813
BOK	1,098387	0,001216	0,023659
SERPINF2	1,098097	0,000719	0,017338
ABHD11-AS1	1,094943	0,000593	0,015373
FOXO4	1,09382	7,76E-05	0,004242
C21orf62	1,09025	2,47E-06	0,00045
LINC01126	1,089976	0,00067	0,016495
CDK18	1,087038	0,001069	0,021902
FAM151A	1,079392	0,002444	0,03647
TMPRSS5	1,073552	0,001215	0,023659
MAMDC4	1,066328	0,001763	0,030035
TSACC	1,065933	0,004239	0,049955
ZIC5	1,064931	0,002712	0,038922
SLC6A9	1,06461	2,65E-07	9,25E-05
SLC38A2	1,063568	1,82E-08	1,68E-05
FZD10-AS1	1,062488	0,002088	0,033123
SNORA25	1,058799	1,69E-06	0,000346
C9orf153	1,05779	0,001124	0,022585
DOCK5	1,05532	0,002969	0,040877
SNORD104	1,049026	0,00064	0,016048

CNTFR-AS1	1,047189	0,003972	0,047902
ASB9P1	1,047172	0,001071	0,021902
KIF1C	1,045642	0,00161	0,028415
MCM7	1,042723	0,000833	0,01865
TSHR	1,042658	0,000196	0,007471
FKBP5	1,041636	1,10E-05	0,00119
S100A4	1,038986	0,000591	0,015373
SLC7A9	1,035951	1,67E-06	0,000346
CLMN	1,035797	0,00203	0,032666
HPN	1,034997	0,003923	0,047549
C16orf71	1,034901	0,000108	0,005224
SNORA38B	1,034454	7,07E-06	0,000911
TLDC2	1,034071	0,000202	0,007639
PLPP2	1,032364	0,003971	0,047902
MGAM	1,029235	0,003488	0,044588
MS4A14	1,027828	0,00248	0,036809
COL8A2	1,023361	0,00029	0,009605
MID1IP1	1,022397	0,000539	0,01443
LOC101928123	1,020028	0,00058	0,015291
HILS1	1,019477	0,000194	0,007465
SMTN	1,017832	0,001023	0,021389
CPM	1,016884	6,99E-06	0,000911
LOC100130548	1,015562	0,001025	0,021396
FAM222A	1,011365	0,000187	0,007296
PCA3	1,009394	0,00082	0,01865
PPFIBP2	1,008969	7,89E-05	0,00428
SNORA26	1,008861	0,000913	0,019888
PRSS8	1,006981	0,000858	0,019041
TJP3	1,006692	2,12E-05	0,001826
SPDYA	1,002663	0,000254	0,008789
RND1	-1,00222	0,002257	0,034742
SOHLH1	-1,00298	0,002334	0,035386
TRIM54	-1,00545	0,00408	0,048674
CCKBR	-1,00821	0,000596	0,015418
TUBB3	-1,00891	1,07E-05	0,001184
SCG2	-1,00923	1,64E-05	0,001546
USP2-AS1	-1,00941	0,000133	0,006023
CPLX1	-1,01124	7,38E-06	0,000929
TPBGL	-1,01223	6,71E-05	0,0039
PCDHGC5	-1,01269	1,11E-07	5,14E-05
IL1RL2	-1,0139	0,000166	0,006786
NLRP14	-1,01429	0,001034	0,021491
DUSP27	-1,01884	0,00034	0,010698
PTPRD-AS2	-1,02078	3,81E-05	0,002704
SYP	-1,02103	0,000173	0,006966
SVOP	-1,02223	5,67E-06	0,000773

RDH12	-1,02336	0,000222	0,008097
GJA5	-1,02381	0,003393	0,043926
CENPH	-1,02443	0,000454	0,013079
KAZALD1	-1,02672	1,88E-05	0,001718
TINAG	-1,03061	0,001645	0,028816
MDH1B	-1,03142	1,64E-06	0,000346
SPTSSB	-1,03249	6,01E-05	0,003671
PTPRQ	-1,03309	0,002855	0,039988
C11orf21	-1,03314	0,003836	0,046999
PAK1	-1,03378	0,000124	0,00575
TRPA1	-1,03522	9,53E-05	0,004788
LINC01119	-1,0353	0,003808	0,046853
TSG1	-1,04262	0,002044	0,032776
CHGB	-1,0433	0,000135	0,006066
COL5A2	-1,04343	0,002633	0,038291
ABCC11	-1,04553	4,80E-06	0,000685
VSNL1	-1,04685	0,000301	0,009779
LOC101928238	-1,05037	3,57E-06	0,000571
LINC00398	-1,05314	0,001915	0,031435
SEMA3G	-1,06486	0,001391	0,025793
ANKRD18DP	-1,06499	2,41E-05	0,001978
RIIAD1	-1,06533	0,000186	0,007296
LINC01336	-1,07518	1,10E-05	0,00119
GCKR	-1,07713	4,94E-06	0,000696
SCN11A	-1,08101	3,23E-06	0,000546
C1QL3	-1,08138	0,002253	0,034733
IL4I1	-1,08389	0,003542	0,04504
SPRED3	-1,08792	1,68E-06	0,000346
THCAT155	-1,08871	1,21E-07	5,48E-05
MSC-AS1	-1,08924	0,000107	0,005224
DHRS11	-1,09659	1,80E-11	5,47E-08
GLP2R	-1,09848	0,003815	0,046853
ADAD2	-1,10138	0,004026	0,048284
WNT1	-1,10386	3,38E-05	0,002511
HHLA2	-1,10425	0,002818	0,039689
KMO	-1,10508	0,000589	0,015373
CRYM	-1,10638	0,000288	0,009583
LINC01605	-1,10681	0,004159	0,04917
LOC340017	-1,11562	0,00389	0,047284
C17orf102	-1,11569	0,000181	0,007171
DUSP5	-1,12048	0,001239	0,023941
SH2D5	-1,12214	9,38E-06	0,001085
LYRM9	-1,12582	2,92E-06	0,000504
RBM3	-1,12907	6,17E-06	0,000831
GCNT4	-1,13399	1,04E-05	0,001168
LOC100129620	-1,13852	0,000361	0,01116

ANGPTL4	-1,16635	0,000586	0,015359
HTR3B	-1,16649	0,001337	0,025071
RAB6C-AS1	-1,16818	0,003044	0,041566
SDS	-1,16868	0,000201	0,007616
PART1	-1,17239	0,00124	0,023941
CITED1	-1,17276	2,91E-05	0,002274
FBLN7	-1,17561	0,000224	0,008135
H19	-1,17732	0,001196	0,023378
ARL4D	-1,17823	8,21E-06	0,000986
PLK4	-1,17823	0,000457	0,013109
LOC101926975	-1,18761	0,000603	0,015468
IQGAP3	-1,19148	0,001354	0,025291
PSG8	-1,19467	0,002943	0,040599
OTOGL	-1,19563	1,92E-05	0,001729
FAM86HP	-1,19565	3,78E-08	2,59E-05
NRN1	-1,1958	6,07E-08	3,59E-05
SPDEF	-1,19894	0,000176	0,007039
PNMA3	-1,19973	7,70E-07	0,000205
LINC01310	-1,20286	0,000797	0,018343
BEX5	-1,20375	8,15E-08	4,13E-05
C3orf80	-1,20824	3,20E-06	0,000544
IGFN1	-1,21511	0,003608	0,045496
PRMT8	-1,21928	2,84E-07	9,45E-05
OR1F1	-1,22029	0,001349	0,02524
LOC105378385	-1,22041	8,42E-05	0,004451
IL33	-1,22434	7,26E-06	0,00092
INHBA-AS1	-1,22657	1,05E-05	0,001171
LINC00368	-1,22848	0,001312	0,024772
HCRTR1	-1,2289	0,001152	0,022888
COLEC10	-1,23334	0,000148	0,006342
LOC101926941	-1,23525	0,000113	0,005403
C9orf106	-1,23703	0,00021	0,007849
LOC339685	-1,24038	0,002247	0,034715
CCDC184	-1,2419	3,81E-06	0,000599
GBP4	-1,24276	0,002728	0,039031
ETV7	-1,24361	0,001853	0,03084
ZBBX	-1,24378	2,13E-05	0,001828
SLC30A3	-1,244	0,002392	0,036015
ARL5C	-1,25515	0,001771	0,030098
GADD45A	-1,2671	0,000107	0,005224
MSC	-1,28199	8,94E-05	0,004651
RFX8	-1,28327	2,33E-05	0,00194
SLC16A6	-1,28412	2,44E-06	0,00045
KCNE2	-1,28892	0,000395	0,011821
LOC101929705	-1,28934	7,08E-05	0,00407
STAT4	-1,2903	6,35E-05	0,003775

GBP6	-1,29058	0,000743	0,017659
ALOX12B	-1,29391	8,40E-06	0,001004
KCNE5	-1,29765	5,19E-08	3,15E-05
PRSS35	-1,30263	0,000105	0,00515
KIRREL3-AS3	-1,309	0,000939	0,020233
FAM86B3P	-1,31808	1,12E-06	0,000256
ATOH7	-1,32566	4,42E-06	0,000667
RAB17	-1,33362	0,002883	0,040115
IFNL1	-1,33695	0,002016	0,032496
OTOG	-1,33922	0,000258	0,008934
MCHR1	-1,34233	1,21E-05	0,001259
LIN28B-AS1	-1,344	0,000938	0,020233
MUC5B	-1,35068	0,000963	0,020565
MROH5	-1,35375	0,000806	0,018475
TAC1	-1,36461	0,000526	0,014304
DHDH	-1,36827	0,001468	0,026802
CD38	-1,38592	0,000508	0,014021
DUSP4	-1,41346	5,49E-05	0,003456
BAAT	-1,43048	0,000301	0,009779
ADRA1D	-1,43084	2,16E-05	0,001839
CD244	-1,45265	0,000512	0,014096
LINC00460	-1,45735	0,003599	0,045457
FREM3	-1,46189	0,000282	0,009472
F3	-1,46562	6,22E-06	0,000832
PCDH8	-1,46595	0,004203	0,049621
LOC339260	-1,47939	0,001777	0,030125
LINC00365	-1,48791	0,001996	0,032306
AQP8	-1,48815	0,000884	0,019495
LINC01007	-1,50007	0,001526	0,027573
SOWAHB	-1,50387	1,34E-07	5,81E-05
BAALC-AS1	-1,50402	1,68E-07	6,48E-05
HEPACAM2	-1,50843	0,003744	0,046475
RGS4	-1,51368	5,08E-05	0,003278
CYP24A1	-1,51793	0,002152	0,033712
KRT81	-1,53343	0,000686	0,016796
RPH3A	-1,55188	2,13E-15	1,51E-11
LOC728084	-1,55876	0,000838	0,01872
SFTPA1	-1,56169	4,47E-06	0,000669
HECTD2-AS1	-1,56338	0,000159	0,006625
RPLP0P2	-1,58059	1,25E-06	0,000284
NKG7	-1,60164	0,000714	0,017264
HOTS	-1,60467	0,002781	0,039441
LINC01107	-1,61242	7,07E-05	0,00407
LINC01168	-1,61824	9,36E-07	0,000229
PPEF1	-1,65707	2,32E-08	1,96E-05
SUCNR1	-1,66616	0,002676	0,038647

ABCC12	-1,67659	5,62E-10	1,32E-06
LINC00343	-1,69013	0,000782	0,018145
HSPB3	-1,69131	8,70E-07	0,00022
C6orf223	-1,6923	2,46E-06	0,00045
NEUROD6	-1,71544	1,75E-09	3,10E-06
LOC102723493	-1,73106	2,48E-05	0,002014
P2RX6P	-1,73426	5,36E-05	0,003412
CALML3	-1,74559	0,002981	0,040991
LINC01164	-1,78659	0,000932	0,02015
LOC100506085	-1,79401	0,002284	0,034848
LOC100506274	-1,83431	0,000157	0,006554
EPHA1-AS1	-1,83702	0,001172	0,023112
PCSK1	-1,8612	5,39E-09	7,64E-06
MIR655	-1,86588	0,003248	0,04284
NAT16	-1,88392	2,80E-09	4,59E-06
LOC101929719	-1,91398	0,000361	0,01116
SLN	-1,95754	0,001836	0,030731
HES5	-1,96634	1,70E-07	6,48E-05
ADCYAP1	-1,97749	1,01E-08	1,15E-05
SLC39A2	-2,01153	0,003909	0,047461
EXOC3L4	-2,0222	0,003305	0,043244
MAS1	-2,05207	4,79E-08	3,09E-05
KRT5	-2,05624	4,63E-06	0,000669
SST	-2,06772	1,18E-05	0,001248
MYBL2	-2,07287	0,00088	0,019424
MPO	-2,07947	1,02E-08	1,15E-05
KLHDC7B	-2,09269	0,00043	0,012607
KIF2B	-2,09776	7,89E-07	0,000207
C2orf48	-2,1299	0,001123	0,022582
LINC01257	-2,24266	7,72E-06	0,000944
LINC01219	-2,2743	3,70E-05	0,002666
LOC401557	-2,27648	0,00174	0,029827
LINC00898	-2,2788	4,66E-06	0,000669
OR7E47P	-2,281	0,00187	0,031029
MUC5AC	-2,28615	0,002553	0,037592
LOC101928505	-2,33222	2,09E-05	0,001826
ELANE	-2,37131	1,61E-06	0,000342
ATP12A	-2,38751	0,000285	0,009539
DLX4	-2,39792	0,00045	0,012989
CSF3	-2,43934	5,51E-05	0,003456
VGF	-2,44636	4,33E-16	9,22E-12
CRH	-2,4555	7,45E-12	2,64E-08
CARTPT	-2,4793	0,000155	0,006554
AHSG	-2,52571	0,000112	0,005373
PRTN3	-2,54461	1,81E-05	0,001676
RFX6	-2,59476	0,000772	0,018014

CLEC4F	-2,61146	0,000518	0,014187
SELE	-2,67458	1,10E-07	5,14E-05
LINC01478	-2,69466	0,002198	0,034182
CDH16	-2,72057	0,003132	0,042226
CHRNB4	-2,794	0,000397	0,011863
SFN	-3,15702	2,16E-05	0,001839
