# Supplementary material for: Berberine Exerts Neuroprotective Effects in Alzheimer’s Disease by Switching Microglia M1/M2 Polarization Through PI3K-AKT Signaling
Source: Physiol Res. 2025 Feb 1;74(1):129–40. doi: 10.33549/physiolres.935410 (PMC11995938; doi:10.33549/physiolres.935410)
Supplement: Supplementary file 2 [file 74_129_TableS2.pdf]

refGene	Modules
AZIN2	lightcyan
STMN1	lightcyan
RAB3B	lightcyan
ITGB3BP	lightcyan
LEPR	lightcyan
PIGK	lightcyan
PKN2-AS1	lightcyan
FAM69A	lightcyan
NBPF9	lightcyan
DNM3	lightcyan
CDC42	lightcyan
RHCE	lightcyan
EPS15	lightcyan
NDC1	lightcyan
LINC01358	lightcyan
DNAJC6	lightcyan
LRRC40	lightcyan
TNNI3K	lightcyan
MIGA1	lightcyan
PRKACB	lightcyan
SAMD13	lightcyan
LRRC8B	lightcyan
HFM1	lightcyan
PTBP2	lightcyan
MFSD14A	lightcyan
VCAM1	lightcyan
STXBP3	lightcyan
SYT6	lightcyan
BCL2L15	lightcyan
WARS2	lightcyan
LINC00623	lightcyan
MSTO1	lightcyan
SYT11	lightcyan
CD244	lightcyan
PFDN2	lightcyan
NUF2	lightcyan
TIPRL	lightcyan
ATP1B1	lightcyan
VAMP4	lightcyan
RABGAP1L	lightcyan
PAPPA2	lightcyan
TSEN15	lightcyan
PLA2G4A	lightcyan
ARL8A	lightcyan
YOD1	lightcyan

SYT14	lightcyan
INTS7	lightcyan
LPGAT1	lightcyan
LYPLAL1-AS1	lightcyan
SRP9	lightcyan
TOMM20	lightcyan
LGALS8	lightcyan
ADSS	lightcyan
ZNF670	lightcyan
ISG15	lightcyan
ENO1-AS1	lightcyan
NPPA	lightcyan
SDHB	lightcyan
PITHD1	lightcyan
FUCA1	lightcyan
TRIM63	lightcyan
PDIK1L	lightcyan
LOC101928728	lightcyan
IFI6	lightcyan
SCARNA1	lightcyan
STX12	lightcyan
SNRNP40	lightcyan
PSMB2	lightcyan
UTP11	lightcyan
PPT1	lightcyan
ZNF684	lightcyan
CITED4	lightcyan
ZMYND12	lightcyan
EBNA1BP2	lightcyan
ATP6V0B	lightcyan
AKR1A1	lightcyan
CYP4A11	lightcyan
LINC00853	lightcyan
BTF3L4	lightcyan
COA7	lightcyan
LOC100507564	lightcyan
TMEM59	lightcyan
DHCR24	lightcyan
ALG6	lightcyan
RPE65	lightcyan
ZRANB2-AS1	lightcyan
CRYZ	lightcyan
CTBS	lightcyan
LOC646626	lightcyan
CLCA3P	lightcyan
SELENOF	lightcyan

GTF2B	lightcyan
KYAT3	lightcyan
GEMIN8P4	lightcyan
GLMN	lightcyan
MIR760	lightcyan
ALG14	lightcyan
LOC101928098	lightcyan
AGL	lightcyan
RTCA	lightcyan
EXTL2	lightcyan
HENMT1	lightcyan
SARS	lightcyan
SORT1	lightcyan
PSMA5	lightcyan
LAMTOR5-AS1	lightcyan
PROK1	lightcyan
LRIF1	lightcyan
CEPT1	lightcyan
ATP5F1	lightcyan
CAPZA1	lightcyan
BCAS2	lightcyan
LOC101928977	lightcyan
WARS2-IT1	lightcyan
HFE2	lightcyan
GNRHR2	lightcyan
HORMAD1	lightcyan
PSMB4	lightcyan
LOC100132111	lightcyan
THEM5	lightcyan
PRR9	lightcyan
S100A9	lightcyan
S100A8	lightcyan
CKS1B	lightcyan
ARHGEF2	lightcyan
TAGLN2	lightcyan
LINC01133	lightcyan
NDUFS2	lightcyan
UHMK1	lightcyan
ALDH9A1	lightcyan
TADA1	lightcyan
LINC01363	lightcyan
CREG1	lightcyan
SELP	lightcyan
F5	lightcyan
PIGC	lightcyan
SNORD81	lightcyan

SNORD47	lightcyan
SNORD44	lightcyan
SNORA103	lightcyan
SNORD76	lightcyan
SNORD75	lightcyan
LINC01645	lightcyan
MIR4424	lightcyan
ZNF648	lightcyan
GLUL	lightcyan
RGS16	lightcyan
ARPC5	lightcyan
PDC	lightcyan
RGS1	lightcyan
LINC01032	lightcyan
UCHL5	lightcyan
B3GALT2	lightcyan
MIR4735	lightcyan
ZNF281	lightcyan
TIMM17A	lightcyan
KLHL12	lightcyan
ADORA1	lightcyan
NUCKS1	lightcyan
C1orf186	lightcyan
IL24	lightcyan
PFKFB2	lightcyan
CR2	lightcyan
SERTAD4	lightcyan
DTL	lightcyan
LINC01740	lightcyan
TATDN3	lightcyan
MIR215	lightcyan
BPNT1	lightcyan
LINC01352	lightcyan
TAF1A-AS1	lightcyan
AIDA	lightcyan
FBXO28	lightcyan
DEGS1	lightcyan
MIR4742	lightcyan
ARV1	lightcyan
TSNAX	lightcyan
MIR1537	lightcyan
ACTN2	lightcyan
FH	lightcyan
SCCPDH	lightcyan
ZNF669	lightcyan
OR2T4	lightcyan

TMEFF2	lightcyan
YIPF4	lightcyan
WTH3DI	lightcyan
ABCB11	lightcyan
OLA1	lightcyan
AGPS	lightcyan
MIR548N	lightcyan
ZSWIM2	lightcyan
XRCC5	lightcyan
TMBIM1	lightcyan
KCNS3	lightcyan
GEN1	lightcyan
APOB	lightcyan
SELENOI	lightcyan
FAM98A	lightcyan
STRN	lightcyan
LRPPRC	lightcyan
SRBD1	lightcyan
RTN4	lightcyan
ETAA1	lightcyan
C1D	lightcyan
CLEC4F	lightcyan
EVA1A	lightcyan
SUCLG1	lightcyan
THNSL2	lightcyan
LINC01103	lightcyan
LOC102724691	lightcyan
LINC01102	lightcyan
C2orf40	lightcyan
BUB1	lightcyan
CKAP2L	lightcyan
C2orf27A	lightcyan
ZRANB3	lightcyan
ORC4	lightcyan
ACVR1C	lightcyan
PSMD14	lightcyan
GCA	lightcyan
SPC25	lightcyan
MYO3B	lightcyan
SESTD1	lightcyan
DNAJC10	lightcyan
WDR75	lightcyan
MFSD6	lightcyan
NABP1	lightcyan
CARF	lightcyan
ABCA12	lightcyan

FARSB	lightcyan
ACSL3	lightcyan
DNER	lightcyan
FBXO36	lightcyan
PSMD1	lightcyan
COL6A3	lightcyan
ACP1	lightcyan
LOC105373352	lightcyan
TMEM18	lightcyan
NRIR	lightcyan
RSAD2	lightcyan
YWHAQ	lightcyan
LOC101929715	lightcyan
DDX1	lightcyan
MYCNOS	lightcyan
SF3B6	lightcyan
AGBL5-AS1	lightcyan
BIRC6-AS2	lightcyan
FEZ2	lightcyan
LOC102723824	lightcyan
C1GALT1C1L	lightcyan
DYNC2LI1	lightcyan
PIGF	lightcyan
CALM2	lightcyan
ERLEC1	lightcyan
MIR3682	lightcyan
LOC100129434	lightcyan
TMEM17	lightcyan
LGALSL	lightcyan
MIR4434	lightcyan
RAB1A	lightcyan
ACTR2	lightcyan
PROKR1	lightcyan
MIR3126	lightcyan
CD207	lightcyan
MCEE	lightcyan
CCT7	lightcyan
MTHFD2	lightcyan
WDR54	lightcyan
DCTN1-AS1	lightcyan
LINC01291	lightcyan
MIR5000	lightcyan
FABP1	lightcyan
LOC101928403	lightcyan
LOC101927070	lightcyan
IL1RL1	lightcyan

LOC100287010	lightcyan
MRPS9	lightcyan
RGPD4	lightcyan
IL1B	lightcyan
IL1A	lightcyan
LOC101060091	lightcyan
LINC01191	lightcyan
INSIG2	lightcyan
NIFK	lightcyan
RAB6C-AS1	lightcyan
RAB6C	lightcyan
POTEF	lightcyan
CCDC115	lightcyan
PLEKHB2	lightcyan
NOC2LP2	lightcyan
MCM6	lightcyan
HNMT	lightcyan
LINC01412	lightcyan
MMADHC	lightcyan
RBM43	lightcyan
STAM2	lightcyan
LOC100144595	lightcyan
LOC100505984	lightcyan
DYNC1I2	lightcyan
CDCA7	lightcyan
SCRN3	lightcyan
LOC100130691	lightcyan
TTC30B	lightcyan
TTC30A	lightcyan
ORMDL1	lightcyan
STAT1	lightcyan
LOC105747689	lightcyan
COQ10B	lightcyan
HSPE1	lightcyan
BZW1	lightcyan
NIF3L1	lightcyan
PPIL3	lightcyan
NDUFB3	lightcyan
SNORD11B	lightcyan
WDR12	lightcyan
NDUFS1	lightcyan
SNORD51	lightcyan
EEF1B2	lightcyan
LANCL1	lightcyan
CCL20	lightcyan
SP140	lightcyan

ECEL1	lightcyan
CHRND	lightcyan
SCARNA5	lightcyan
COPS8	lightcyan
LOC93463	lightcyan
CLASP2	lightcyan
CMSS1	lightcyan
LINC00882	lightcyan
LOC105374060	lightcyan
COPG1	lightcyan
PIK3CB	lightcyan
KCNAB1	lightcyan
FANCD2	lightcyan
UBE2E1	lightcyan
GADL1	lightcyan
SNORA62	lightcyan
SACM1L	lightcyan
POC1A	lightcyan
PTPRG-AS1	lightcyan
RYBP	lightcyan
CHMP2B	lightcyan
CEP97	lightcyan
LINC00636	lightcyan
CD200	lightcyan
IGSF11	lightcyan
MUC13	lightcyan
SNX4	lightcyan
MRPL3	lightcyan
NPHP3-ACAD11	lightcyan
PPP2R3A	lightcyan
DBR1	lightcyan
HLTF	lightcyan
SELENOT	lightcyan
DHX36	lightcyan
LINC00886	lightcyan
PTX3	lightcyan
IFT80	lightcyan
OTOL1	lightcyan
LINC01324	lightcyan
SERPINI1	lightcyan
SLC7A14	lightcyan
TMEM212	lightcyan
ECT2	lightcyan
NLGN1	lightcyan
NAALADL2-AS2	lightcyan
PEX5L	lightcyan

USP13	lightcyan
LOC102724604	lightcyan
IL1RAP	lightcyan
OSTN	lightcyan
OPA1	lightcyan
ARL8B	lightcyan
LINC00312	lightcyan
LSM3	lightcyan
SLC6A6	lightcyan
RAB5A	lightcyan
MIR4442	lightcyan
OXSM	lightcyan
RBMS3-AS1	lightcyan
DYNC1LI1	lightcyan
EPM2AIP1	lightcyan
EIF1B	lightcyan
SS18L2	lightcyan
HIGD1A	lightcyan
KRBOX1	lightcyan
KIAA1143	lightcyan
UQCRC1	lightcyan
NAT6	lightcyan
IQCF4	lightcyan
SPCS1	lightcyan
ARF4	lightcyan
ABHD6	lightcyan
PDHB	lightcyan
LMOD3	lightcyan
UBA3	lightcyan
ARL6IP5	lightcyan
SAMMSON	lightcyan
ZNF717	lightcyan
CGGBP1	lightcyan
NSUN3	lightcyan
STX19	lightcyan
GPR15	lightcyan
NIT2	lightcyan
TOMM70	lightcyan
TRMT10C	lightcyan
ZPLD1	lightcyan
CD47	lightcyan
HHLA2	lightcyan
KIAA1524	lightcyan
PVRL3-AS1	lightcyan
ABHD10	lightcyan
ATG3	lightcyan

LINC01279	lightcyan
SLC35A5	lightcyan
CD200R1	lightcyan
LOC101929717	lightcyan
CFAP44-AS1	lightcyan
NDUFB4	lightcyan
CASR	lightcyan
CCDC58	lightcyan
WDR5B	lightcyan
FAM162A	lightcyan
HACD2	lightcyan
ALG1L	lightcyan
ALG1L2	lightcyan
ANAPC13	lightcyan
PCCB	lightcyan
RBP1	lightcyan
SLC25A36	lightcyan
LOC100289361	lightcyan
CPB1	lightcyan
TM4SF1	lightcyan
LINC01213	lightcyan
ERICH6-AS1	lightcyan
SUCNR1	lightcyan
KCNAB1-AS1	lightcyan
TIPARP	lightcyan
LOC100996447	lightcyan
IL12A	lightcyan
PDCD10	lightcyan
LRRC34	lightcyan
GPR160	lightcyan
RPL22L1	lightcyan
EIF5A2	lightcyan
NAALADL2-AS1	lightcyan
NDUFB5	lightcyan
LOC100505609	lightcyan
DNAJC19	lightcyan
SNORD2	lightcyan
SNORA63	lightcyan
KNG1	lightcyan
SST	lightcyan
TMEM207	lightcyan
UTS2B	lightcyan
OPA1-AS1	lightcyan
LRRC15	lightcyan
PPP1R2	lightcyan
FAM157A	lightcyan

LIN54	lightcyan
CISD2	lightcyan
C4orf33	lightcyan
TRIM2	lightcyan
MFAP3L	lightcyan
LOC101928306	lightcyan
RFC1	lightcyan
SMIM14	lightcyan
RHOH	lightcyan
SLC30A9	lightcyan
ATP8A1	lightcyan
SCFD2	lightcyan
SRD5A3	lightcyan
EXOC1	lightcyan
LOC401134	lightcyan
TMPRSS11F	lightcyan
COX18	lightcyan
RCHY1	lightcyan
FAM47E	lightcyan
ENOPH1	lightcyan
TSPAN5	lightcyan
RAP1GDS1	lightcyan
MTTP	lightcyan
ADH1C	lightcyan
CENPE	lightcyan
LOC101929468	lightcyan
OSTC	lightcyan
MCUB	lightcyan
RRH	lightcyan
ELOVL6	lightcyan
NDST4	lightcyan
MFSD8	lightcyan
SCOC	lightcyan
KLHL2	lightcyan
HPF1	lightcyan
LOC101928551	lightcyan
CFAP97	lightcyan
ZNF595	lightcyan
TMEM128	lightcyan
LOC105374366	lightcyan
LOC93622	lightcyan
FAM200B	lightcyan
CD38	lightcyan
LAP3	lightcyan
DCAF16	lightcyan
MIR218-1	lightcyan

SHISA3	lightcyan
COMMD8	lightcyan
OCIAD1	lightcyan
SGCB	lightcyan
SRP72	lightcyan
POLR2B	lightcyan
GNRHR	lightcyan
RUFY3	lightcyan
DCK	lightcyan
CXCL8	lightcyan
CXCL1	lightcyan
CXCL3	lightcyan
CXCL10	lightcyan
SCARB2	lightcyan
RASGEF1B	lightcyan
SNORD143	lightcyan
SNORD144	lightcyan
THAP9-AS1	lightcyan
COPS4	lightcyan
COQ2	lightcyan
C4orf36	lightcyan
HSD17B11	lightcyan
IBSP	lightcyan
TIGD2	lightcyan
TRMT10A	lightcyan
DNAJB14	lightcyan
MIR1255A	lightcyan
CXXC4	lightcyan
LOC101929529	lightcyan
PAPSS1	lightcyan
SEC24B-AS1	lightcyan
C4orf32	lightcyan
AP1AR	lightcyan
TIFA	lightcyan
MIR367	lightcyan
MIR302A	lightcyan
MIR1243	lightcyan
MIR8082	lightcyan
TRAM1L1	lightcyan
MAD2L1	lightcyan
TNIP3	lightcyan
CETN4P	lightcyan
ANKRD50	lightcyan
PLK4	lightcyan
LOC101927359	lightcyan
SCOC-AS1	lightcyan

ABCE1	lightcyan
MMAA	lightcyan
FGG	lightcyan
LOC102724776	lightcyan
CTSO	lightcyan
C4orf46	lightcyan
TMA16	lightcyan
FAM218A	lightcyan
MSMO1	lightcyan
CLCN3	lightcyan
SPCS3	lightcyan
AGA	lightcyan
UFSP2	lightcyan
OSMR-AS1	lightcyan
IPO11	lightcyan
ANKRD31	lightcyan
LIX1	lightcyan
SLC25A46	lightcyan
FNIP1	lightcyan
SPOCK1	lightcyan
FASTKD3	lightcyan
LOC285692	lightcyan
FAM134B	lightcyan
RICTOR	lightcyan
C9	lightcyan
C6	lightcyan
DEPDC1B	lightcyan
NDUFAF2	lightcyan
GFM2	lightcyan
SCAMP1	lightcyan
ATG10	lightcyan
XRCC4	lightcyan
TMEM161B	lightcyan
ELL2	lightcyan
LINC01340	lightcyan
FAM174A	lightcyan
NUDT12	lightcyan
COMMD10	lightcyan
LOC101927460	lightcyan
FBN2	lightcyan
CDC42SE2	lightcyan
SAR1B	lightcyan
C5orf15	lightcyan
ANKHD1	lightcyan
SPINK5	lightcyan
ATOX1	lightcyan

FABP6	lightcyan
FAM196B	lightcyan
RANBP17	lightcyan
STC2	lightcyan
RASGEF1C	lightcyan
SDHA	lightcyan
MTRR	lightcyan
MIR4458HG	lightcyan
TAS2R1	lightcyan
CCT5	lightcyan
C5orf22	lightcyan
MIR579	lightcyan
AMACR	lightcyan
MIR580	lightcyan
NIPBL-AS1	lightcyan
OXCT1-AS1	lightcyan
C5orf51	lightcyan
HMGCS1	lightcyan
TMEM267	lightcyan
C5orf34	lightcyan
MOCS2	lightcyan
ESM1	lightcyan
DIMT1	lightcyan
LRRC70	lightcyan
TRIM23	lightcyan
TRAPPC13	lightcyan
NLN	lightcyan
CENPH	lightcyan
MRPS36	lightcyan
TAF9	lightcyan
AK6	lightcyan
ANKRA2	lightcyan
NSA2	lightcyan
LOC441086	lightcyan
F2RL1	lightcyan
SCAMP1-AS1	lightcyan
CRSP8P	lightcyan
LOC644936	lightcyan
RASGRF2-AS1	lightcyan
TMEM167A	lightcyan
SCARNA18	lightcyan
COX7C	lightcyan
LOC731157	lightcyan
MBLAC2	lightcyan
LYSMD3	lightcyan
ARRDC3	lightcyan

RFESD	lightcyan
CTD-2151A2.1	lightcyan
ST8SIA4	lightcyan
LOC102467212	lightcyan
PJA2	lightcyan
WDR36	lightcyan
REEP5	lightcyan
ISOC1	lightcyan
HINT1	lightcyan
SKP1	lightcyan
PPP2CA	lightcyan
MIR4461	lightcyan
TIFAB	lightcyan
DCANP1	lightcyan
LRRTM2	lightcyan
SNORA74A	lightcyan
PAIP2	lightcyan
LOC101929719	lightcyan
PCDHB2	lightcyan
LOC101926905	lightcyan
YIPF5	lightcyan
GRPEL2	lightcyan
SLC26A2	lightcyan
DCTN4	lightcyan
IRGM	lightcyan
PPP1R2P3	lightcyan
SOX30	lightcyan
UBLCP1	lightcyan
HMMR	lightcyan
MAT2B	lightcyan
MIR103A1	lightcyan
NPM1	lightcyan
MIR1271	lightcyan
LOC728554	lightcyan
LOC100996419	lightcyan
CAGE1	lightcyan
F13A1	lightcyan
DNAH8	lightcyan
TTK	lightcyan
AMD1	lightcyan
NKAIN2	lightcyan
PACRG	lightcyan
IRF4	lightcyan
LOC101928519	lightcyan
LOC101929163	lightcyan
GLO1	lightcyan

LMBRD1	lightcyan
FILIP1	lightcyan
ORC3	lightcyan
SLC35A1	lightcyan
UFL1	lightcyan
CCNC	lightcyan
LOC100996634	lightcyan
PKIB	lightcyan
NCOA7	lightcyan
ECHDC1	lightcyan
PTPRK	lightcyan
THEMIS	lightcyan
TMEM244	lightcyan
EYA4	lightcyan
TARID	lightcyan
GVQW2	lightcyan
VTA1	lightcyan
PCMT1	lightcyan
GTF2H5	lightcyan
SOD2	lightcyan
AGPAT4	lightcyan
PDE10A	lightcyan
KIF25	lightcyan
MIR4645	lightcyan
LOC100507194	lightcyan
RPP40	lightcyan
EEF1E1	lightcyan
C6orf52	lightcyan
SMIM13	lightcyan
ADTRP	lightcyan
LOC101928433	lightcyan
NHLRC1	lightcyan
HIST1H2AD	lightcyan
HIST1H2BD	lightcyan
HIST1H2BF	lightcyan
HIST1H2BC	lightcyan
HIST1H1E	lightcyan
HIST1H2BG	lightcyan
HIST1H2BI	lightcyan
HIST1H3E	lightcyan
HIST1H2BK	lightcyan
HIST1H2AH	lightcyan
HIST1H2BJ	lightcyan
HIST1H4K	lightcyan
ZSCAN12P1	lightcyan
LINC00533	lightcyan

LINC01556	lightcyan
HLA-C	lightcyan
SNORD117	lightcyan
C2	lightcyan
HCG23	lightcyan
HLA-DQB1	lightcyan
LOC100294145	lightcyan
TCP11	lightcyan
CLPSL1	lightcyan
PANDAR	lightcyan
OARD1	lightcyan
TREM1	lightcyan
TBCC	lightcyan
MAD2L1BP	lightcyan
ENPP4	lightcyan
LOC101926898	lightcyan
TMEM14A	lightcyan
ELOVL5	lightcyan
KLHL31	lightcyan
KIAA1586	lightcyan
GUSBP4	lightcyan
PTP4A1	lightcyan
LOC441155	lightcyan
LOC101928307	lightcyan
EVADR	lightcyan
SDHAF4	lightcyan
COX7A2	lightcyan
TMEM30A	lightcyan
IBTK	lightcyan
SNORD50A	lightcyan
SNORD50B	lightcyan
MIR2113	lightcyan
POPDC3	lightcyan
LINC01268	lightcyan
RWDD1	lightcyan
RSPH4A	lightcyan
ASF1A	lightcyan
HSF2	lightcyan
SERINC1	lightcyan
LOC643623	lightcyan
TAAR6	lightcyan
TAAR5	lightcyan
LINC00326	lightcyan
LINC01312	lightcyan
HBS1L	lightcyan
BCLAF1	lightcyan

LINC01277	lightcyan
FUCA2	lightcyan
ZC2HC1B	lightcyan
LRP11	lightcyan
ACAT2	lightcyan
T	lightcyan
SFT2D1	lightcyan
LOC100289495	lightcyan
MPC1	lightcyan
TTLL2	lightcyan
LOC441178	lightcyan
LOC101929460	lightcyan
SRPK2	lightcyan
NAMPT	lightcyan
RPA3	lightcyan
LOC101927391	lightcyan
RAPGEF5	lightcyan
FAM221A	lightcyan
SKAP2	lightcyan
07.IX	lightcyan
ELMO1	lightcyan
DDC	lightcyan
SBDS	lightcyan
SEMA3C	lightcyan
GNAI1	lightcyan
LOC101927356	lightcyan
SEMA3A	lightcyan
SEMA3E	lightcyan
LINC00972	lightcyan
GRM3	lightcyan
CYP51A1	lightcyan
DUS4L	lightcyan
CAPZA2	lightcyan
NDUFA5	lightcyan
POT1	lightcyan
BPGM	lightcyan
WEE2-AS1	lightcyan
TPK1	lightcyan
LINC01006	lightcyan
ACTB	lightcyan
CCZ1B	lightcyan
TMEM106B	lightcyan
AHR	lightcyan
TWISTNB	lightcyan
LOC101927811	lightcyan
STEAP1B	lightcyan

KLHL7-AS1	lightcyan
CLK2P1	lightcyan
CCDC126	lightcyan
NPY	lightcyan
DPY19L2P3	lightcyan
LOC105375218	lightcyan
PPP1R17	lightcyan
NT5C3A	lightcyan
PSMA2	lightcyan
PPIA	lightcyan
MYO1G	lightcyan
C7orf65	lightcyan
FIGNL1	lightcyan
SEC61G	lightcyan
GBAS	lightcyan
CCT6A	lightcyan
CHCHD2	lightcyan
ZNF727	lightcyan
YWHAEP1	lightcyan
ZNF138	lightcyan
CCT6P3	lightcyan
ZNF92	lightcyan
NSUN5	lightcyan
LOC101926943	lightcyan
GTF2IP7	lightcyan
LOC101927243	lightcyan
TMEM60	lightcyan
LOC101927420	lightcyan
SLC25A40	lightcyan
SRI	lightcyan
GTPBP10	lightcyan
HEPACAM2	lightcyan
TFPI2	lightcyan
CASD1	lightcyan
SDHAF3	lightcyan
MIR5692C2	lightcyan
NPTX2	lightcyan
OR2AE1	lightcyan
GJC3	lightcyan
FIS1	lightcyan
LRRC17	lightcyan
ARMC10	lightcyan
PSMC2	lightcyan
BCAP29	lightcyan
CBLL1	lightcyan
DLD	lightcyan

DNAJB9	lightcyan
THAP5	lightcyan
EIF3IP1	lightcyan
TMEM168	lightcyan
LINC00998	lightcyan
ANKRD7	lightcyan
LOC101928211	lightcyan
C7orf77	lightcyan
HILPDA	lightcyan
TPI1P2	lightcyan
TMEM209	lightcyan
CEP41	lightcyan
MIR335	lightcyan
FMC1	lightcyan
JHDM1D-AS1	lightcyan
MRPS33	lightcyan
CTAGE4	lightcyan
LOC105375556	lightcyan
INSIG1	lightcyan
LINC00244	lightcyan
SLC26A7	lightcyan
PPP2R2A	lightcyan
ZFHX4-AS1	lightcyan
TRHR	lightcyan
MTMR7	lightcyan
PEBP4	lightcyan
EBF2	lightcyan
ESCO2	lightcyan
DCTN6	lightcyan
POLB	lightcyan
MCM4	lightcyan
LOC101929268	lightcyan
RAB2A	lightcyan
CYP7B1	lightcyan
ADHFE1	lightcyan
SULF1	lightcyan
LACTB2	lightcyan
TPD52	lightcyan
IMPA1	lightcyan
NECAB1	lightcyan
CDH17	lightcyan
LOC101927066	lightcyan
LAPTM4B	lightcyan
COLEC10	lightcyan
DSCC1	lightcyan
MTBP	lightcyan

WASHC5	lightcyan
CASC8	lightcyan
FAM49B	lightcyan
LOC100288181	lightcyan
OR4F21	lightcyan
DEFB109P1B	lightcyan
MTMR9	lightcyan
FAM66D	lightcyan
USP17L7	lightcyan
FGF20	lightcyan
CNOT7	lightcyan
LOC101929066	lightcyan
HR	lightcyan
LGI3	lightcyan
BNIP3L	lightcyan
SARAF	lightcyan
LEPROTL1	lightcyan
MAK16	lightcyan
DDHD2	lightcyan
TM2D2	lightcyan
VDAC3	lightcyan
HGSNAT	lightcyan
UBE2V2	lightcyan
LYPLA1	lightcyan
SNORD54	lightcyan
PLAG1	lightcyan
MOS	lightcyan
CHCHD7	lightcyan
IMPAD1	lightcyan
ARMC1	lightcyan
C8orf46	lightcyan
COPS5	lightcyan
LOC101926892	lightcyan
XKR9	lightcyan
C8orf89	lightcyan
ELOC	lightcyan
LY96	lightcyan
CRISPLD1	lightcyan
MIR3149	lightcyan
FABP12	lightcyan
ZFAND1	lightcyan
SNX16	lightcyan
C8orf59	lightcyan
LINC01030	lightcyan
OTUD6B-AS1	lightcyan
TRIQK	lightcyan

COX6C	lightcyan
FBXO43	lightcyan
POLR2K	lightcyan
ANKRD46	lightcyan
RRM2B	lightcyan
AZIN1	lightcyan
SLC25A32	lightcyan
EMC2	lightcyan
NUDCD1	lightcyan
ENY2	lightcyan
RAD21	lightcyan
AARD	lightcyan
MRPL13	lightcyan
C8orf76	lightcyan
WDYHV1	lightcyan
TATDN1	lightcyan
SQLE	lightcyan
MYC	lightcyan
NCRNA00250	lightcyan
PTPRD	lightcyan
LINC01230	lightcyan
ADAMTSL1	lightcyan
PPP6C	lightcyan
CBWD1	lightcyan
PLGRKT	lightcyan
SLC24A2	lightcyan
ZNF658	lightcyan
C9orf135	lightcyan
KLF9	lightcyan
OSTF1	lightcyan
IARS	lightcyan
FSD1L	lightcyan
FKTN	lightcyan
PALM2	lightcyan
FAM225B	lightcyan
SNX30	lightcyan
GGTA1P	lightcyan
PSMB7	lightcyan
AK8	lightcyan
VLDLR	lightcyan
CD274	lightcyan
RANBP6	lightcyan
TMEM261	lightcyan
TYRP1	lightcyan
LURAP1L	lightcyan
PLIN2	lightcyan

RRAGA	lightcyan
KLHL9	lightcyan
PLAA	lightcyan
C9orf72	lightcyan
NDUFB6	lightcyan
CHMP5	lightcyan
VCP	lightcyan
ATP8B5P	lightcyan
RMRP	lightcyan
HINT2	lightcyan
EBLN3P	lightcyan
ALDH1B1	lightcyan
FGF7P3	lightcyan
PTGER4P2-CDK2AP2P2	lightcyan
FRG1JP	lightcyan
MIR1299	lightcyan
PGM5P2	lightcyan
NMRK1	lightcyan
LOC105376114	lightcyan
MIR7-1	lightcyan
LOC101927575	lightcyan
LOC494127	lightcyan
MIRLET7A1	lightcyan
MRPL50	lightcyan
SMC2-AS1	lightcyan
SMC2	lightcyan
LOC105376194	lightcyan
NIPSNAP3A	lightcyan
FAM206A	lightcyan
TXN	lightcyan
ZFP37	lightcyan
ZNF883	lightcyan
RBM18	lightcyan
OR1N1	lightcyan
OR1L8	lightcyan
OR1B1	lightcyan
OR1L4	lightcyan
OR1L6	lightcyan
PDCL	lightcyan
ZBTB6	lightcyan
HSPA5	lightcyan
ENDOG	lightcyan
TMEM8C	lightcyan
OBP2A	lightcyan
HNRNPH2	lightcyan
CXorf57	lightcyan

GPM6B	lightcyan
PIR	lightcyan
AP1S2	lightcyan
REPS2	lightcyan
MBTPS2	lightcyan
OTC	lightcyan
FAM104B	lightcyan
SPIN2B	lightcyan
MTMR8	lightcyan
CHIC1	lightcyan
MIR325HG	lightcyan
SH3BGRL	lightcyan
POF1B	lightcyan
APOOL	lightcyan
KLHL4	lightcyan
NUP62CL	lightcyan
ACSL4	lightcyan
LINC00890	lightcyan
PLS3	lightcyan
STK26	lightcyan
RAP2C-AS1	lightcyan
PHF6	lightcyan
ZNF449	lightcyan
CLCN4	lightcyan
TMSB4X	lightcyan
GEMIN8	lightcyan
UBE2E4P	lightcyan
VEGFD	lightcyan
PIGA	lightcyan
BMX	lightcyan
TMEM27	lightcyan
SCML1	lightcyan
EIF1AX	lightcyan
PRDX4	lightcyan
DCAF8L2	lightcyan
DCAF8L1	lightcyan
GK	lightcyan
LINC01282	lightcyan
ATP6AP2	lightcyan
MED14	lightcyan
FUNDC1	lightcyan
TIMM17B	lightcyan
PRAF2	lightcyan
MIR98	lightcyan
MAGEH1	lightcyan
RRAGB	lightcyan

ASB12	lightcyan
RLIM	lightcyan
MIR384	lightcyan
TAF9B	lightcyan
PABPC5	lightcyan
PABPC5-AS1	lightcyan
NXF5	lightcyan
TMSB15A	lightcyan
ARMCX5	lightcyan
BEX4	lightcyan
TCEAL7	lightcyan
PRPS1	lightcyan
PSMD10	lightcyan
IRS4	lightcyan
PGRMC1	lightcyan
ZBTB33	lightcyan
C1GALT1C1	lightcyan
XPNPEP2	lightcyan
RAB33A	lightcyan
MIR503	lightcyan
MOSPD1	lightcyan
MMGT1	lightcyan
RBMX	lightcyan
VMA21	lightcyan
NSDHL	lightcyan
LOC105373378	lightcyan
IDH3G	lightcyan
CH17-340M24.3	lightcyan
ATP6AP1	lightcyan
F8A1	lightcyan
VBP1	lightcyan
RAB39B	lightcyan
NLGN4Y	lightcyan
PCDH11Y	lightcyan
LINC00278	lightcyan
PRKY	lightcyan
UTY	lightcyan
USP9Y	lightcyan
NLGN4Y-AS1	lightcyan
KDM5D	lightcyan
TXLNGY	lightcyan
TTTY14	lightcyan
TTTY10	lightcyan
RPS4Y1	lightcyan
ZFY	lightcyan
TTTY15	lightcyan

DDX3Y	lightcyan
TMSB4Y	lightcyan
EIF1AY	lightcyan
ANK3	lightcyan
KIF11	lightcyan
ACADSB	lightcyan
STAM	lightcyan
SPAG6	lightcyan
LOC100130992	lightcyan
LINC01516	lightcyan
MAPK8	lightcyan
PHYHIPL	lightcyan
LINC00845	lightcyan
C10orf107	lightcyan
ARID5B	lightcyan
ZNF365	lightcyan
LRRTM3	lightcyan
ATAD1	lightcyan
TLL2	lightcyan
TM9SF3	lightcyan
SLC18A2	lightcyan
OAT	lightcyan
IDI1	lightcyan
GDI2	lightcyan
ATP5C1	lightcyan
CDC123	lightcyan
ARL5B	lightcyan
COMMD3	lightcyan
LOC100499489	lightcyan
THNSL1	lightcyan
RAB18	lightcyan
LOC101929431	lightcyan
CUL2	lightcyan
ZNF33BP1	lightcyan
LOC100129055	lightcyan
ZNF32-AS3	lightcyan
HNRNPA3P1	lightcyan
OR13A1	lightcyan
FAM35DP	lightcyan
TIMM23	lightcyan
LINC01468	lightcyan
UBE2D1	lightcyan
CDK1	lightcyan
LOC283045	lightcyan
ADO	lightcyan
NRBF2	lightcyan

SNORD98	lightcyan
KIF1BP	lightcyan
PPA1	lightcyan
ANXA7	lightcyan
PPIF	lightcyan
EIF5AL1	lightcyan
SFTPA2	lightcyan
HOST2	lightcyan
CDHR1	lightcyan
MIR4678	lightcyan
LIPJ	lightcyan
CH25H	lightcyan
IFIT1	lightcyan
IFIT5	lightcyan
ANKRD1	lightcyan
XLOC_008559	lightcyan
CYP26A1	lightcyan
SLC35G1	lightcyan
DNTT	lightcyan
LOC102723665	lightcyan
CUTC	lightcyan
SNORA12	lightcyan
BLOC1S2	lightcyan
USMG5	lightcyan
GSTO1	lightcyan
BBIP1	lightcyan
PDCD4-AS1	lightcyan
KCNK18	lightcyan
PRDX3	lightcyan
PLEKHA1	lightcyan
FAM24B	lightcyan
IKZF5	lightcyan
BUB3	lightcyan
MIR4484	lightcyan
CLRN3	lightcyan
BNIP3	lightcyan
C11orf58	lightcyan
TMEM151A	lightcyan
RAB6A	lightcyan
NLRP14	lightcyan
CAND1.11	lightcyan
PSMA1	lightcyan
BDNF-AS	lightcyan
HSD17B12	lightcyan
DDB1	lightcyan
SLC36A4	lightcyan

NCAM1	lightcyan
PAFAH1B2	lightcyan
SIAE	lightcyan
PSMD13	lightcyan
IFITM1	lightcyan
RNH1	lightcyan
LOC143666	lightcyan
TALDO1	lightcyan
ASCL2	lightcyan
RRM1	lightcyan
OR52N5	lightcyan
OR52N1	lightcyan
ZNF214	lightcyan
LOC101928008	lightcyan
CSNK2A3	lightcyan
FAR1	lightcyan
TPH1	lightcyan
LDHA	lightcyan
UEVLD	lightcyan
ZDHHC13	lightcyan
LINC01495	lightcyan
SVIP	lightcyan
EIF3M	lightcyan
FBXO3	lightcyan
EHF	lightcyan
API5	lightcyan
ARHGAP1	lightcyan
ACP2	lightcyan
MTCH2	lightcyan
TIMM10	lightcyan
CLP1	lightcyan
OR5B12	lightcyan
OR5B21	lightcyan
TMEM258	lightcyan
FEN1	lightcyan
FADS1	lightcyan
SNHG1	lightcyan
SNORD30	lightcyan
POLR2G	lightcyan
HRASLS5	lightcyan
CFL1	lightcyan
FAM86C2P	lightcyan
C11orf24	lightcyan
MRPL21	lightcyan
FLJ42102	lightcyan
DEFB108B	lightcyan

LIPT2	lightcyan
KLHL35	lightcyan
NDUFC2	lightcyan
LOC101928865	lightcyan
DDIAS	lightcyan
TMEM126B	lightcyan
RAB38	lightcyan
SNORD6	lightcyan
SNORA8	lightcyan
SNORD5	lightcyan
KDM4D	lightcyan
MTMR2	lightcyan
BIRC3	lightcyan
MMP12	lightcyan
KBTBD3	lightcyan
AASDHPPT	lightcyan
C11orf88	lightcyan
FDXACB1	lightcyan
C11orf1	lightcyan
SDHD	lightcyan
PTS	lightcyan
NCAM1-AS1	lightcyan
C11orf71	lightcyan
SC5D	lightcyan
STT3A	lightcyan
LOC101929497	lightcyan
FAR2	lightcyan
SLC11A2	lightcyan
KLRA1P	lightcyan
ERP27	lightcyan
DERA	lightcyan
SLC15A5	lightcyan
RECQL	lightcyan
ETNK1	lightcyan
BCAT1	lightcyan
DDX11-AS1	lightcyan
AMN1	lightcyan
SLC38A1	lightcyan
PCED1B	lightcyan
C12orf54	lightcyan
SLC4A8	lightcyan
SLC16A7	lightcyan
PPM1H	lightcyan
TBK1	lightcyan
LOC100507250	lightcyan
GLIPR1L1	lightcyan

ZDHHC17	lightcyan
LOC105369921	lightcyan
USP44	lightcyan
CFAP54	lightcyan
UHRF1BP1L	lightcyan
HCFC2	lightcyan
C12orf76	lightcyan
ARPC3	lightcyan
LOC101927592	lightcyan
CACNA1C-IT2	lightcyan
RHNO1	lightcyan
C12orf4	lightcyan
RAD51AP1	lightcyan
NDUFA9	lightcyan
VAMP1	lightcyan
SCARNA10	lightcyan
USP5	lightcyan
KLRF1	lightcyan
LOH12CR2	lightcyan
LINC01559	lightcyan
C12orf60	lightcyan
SMCO3	lightcyan
SKP1P2	lightcyan
PYROXD1	lightcyan
GOLT1B	lightcyan
LINC00477	lightcyan
ETFRF1	lightcyan
RASSF8-AS1	lightcyan
MED21	lightcyan
KLHL42	lightcyan
ERGIC2	lightcyan
DNM1L	lightcyan
ALG10	lightcyan
ALG10B	lightcyan
RPAP3	lightcyan
TUBA1C	lightcyan
TUBA1A	lightcyan
PRPH	lightcyan
LETMD1	lightcyan
DAZAP2	lightcyan
KRT1	lightcyan
KRT2	lightcyan
GPR84	lightcyan
GTSF1	lightcyan
NEUROD4	lightcyan
ATP5B	lightcyan

SNORD59A	lightcyan
HSD17B6	lightcyan
METTL1	lightcyan
C12orf66	lightcyan
TMBIM4	lightcyan
CAND1	lightcyan
IL26	lightcyan
YEATS4	lightcyan
LOC101928137	lightcyan
ATXN7L3B	lightcyan
BBS10	lightcyan
LOC102724663	lightcyan
SLC6A15	lightcyan
TSPAN19	lightcyan
C12orf29	lightcyan
TMTC3	lightcyan
LUM	lightcyan
UBE2N	lightcyan
MRPL42	lightcyan
NDUFA12	lightcyan
SLC25A3	lightcyan
ACTR6	lightcyan
ARL1	lightcyan
SPIC	lightcyan
WASHC3	lightcyan
PARPBP	lightcyan
NFYB	lightcyan
PWP1	lightcyan
MMAB	lightcyan
VPS29	lightcyan
MAPKAPK5-AS1	lightcyan
MIR3657	lightcyan
SDSL	lightcyan
MIR620	lightcyan
PEBP1	lightcyan
PXN-AS1	lightcyan
TRIAP1	lightcyan
TMED2	lightcyan
RAN	lightcyan
ZNF10	lightcyan
PCDH9	lightcyan
KPNA3	lightcyan
TEX26-AS1	lightcyan
DLEU7	lightcyan
LINC00333	lightcyan
TPTE2	lightcyan

MTMR6	lightcyan
USP12	lightcyan
USPL1	lightcyan
RFC3	lightcyan
LINC00457	lightcyan
UFM1	lightcyan
AKAP11	lightcyan
DNAJC15	lightcyan
CCDC122	lightcyan
NDFIP2	lightcyan
MBNL2	lightcyan
LINC00327	lightcyan
POMP	lightcyan
ALG5	lightcyan
EXOSC8	lightcyan
CSNK1A1L	lightcyan
MIR4305	lightcyan
KBTBD6	lightcyan
VWA8-AS1	lightcyan
KCTD4	lightcyan
ESD	lightcyan
SUCLA2	lightcyan
EBPL	lightcyan
LOC101926951	lightcyan
MZT1	lightcyan
LINC00347	lightcyan
CTAGE11P	lightcyan
RNF219	lightcyan
RBM26-AS1	lightcyan
GPR180	lightcyan
LINC00557	lightcyan
MIR623	lightcyan
TEX30	lightcyan
LIG4	lightcyan
MIR4502	lightcyan
PSMA3	lightcyan
STXBP6	lightcyan
SCFD1	lightcyan
G2E3	lightcyan
NUBPL	lightcyan
AKAP6	lightcyan
EAPP	lightcyan
PSMA6	lightcyan
SLC25A21	lightcyan
FANCM	lightcyan
FRMD6-AS2	lightcyan

GPR137C	lightcyan
WDHD1	lightcyan
MPP5	lightcyan
ERH	lightcyan
TMED10	lightcyan
ADCK1	lightcyan
GTF2A1	lightcyan
EFCAB11	lightcyan
LGMN	lightcyan
AK7	lightcyan
LOC101929241	lightcyan
EML1	lightcyan
PARP2	lightcyan
ECRP	lightcyan
DAD1	lightcyan
REM2	lightcyan
DHRS4-AS1	lightcyan
MDP1	lightcyan
NEDD8	lightcyan
LINC00645	lightcyan
LOC100506071	lightcyan
DTD2	lightcyan
CFL2	lightcyan
SRP54	lightcyan
PPP2R3C	lightcyan
SLC25A21-AS1	lightcyan
TRAPPC6B	lightcyan
KLHL28	lightcyan
C14orf28	lightcyan
MIS18BP1	lightcyan
ATL1	lightcyan
LINC00640	lightcyan
C14orf166	lightcyan
PSMC6	lightcyan
CNIH1	lightcyan
GMFB	lightcyan
AP5M1	lightcyan
ACTR10	lightcyan
JKAMP	lightcyan
MIR5586	lightcyan
CHURC1	lightcyan
FNTB	lightcyan
LINC00238	lightcyan
EIF2S1	lightcyan
ACOT1	lightcyan
LOC100506498	lightcyan

SLIRP	lightcyan
LOC101928504	lightcyan
SEL1L	lightcyan
GALC	lightcyan
NDUFB1	lightcyan
DDX24	lightcyan
SERPINA3	lightcyan
BDKRB2	lightcyan
GSKIP	lightcyan
LOC730202	lightcyan
WARS	lightcyan
SNORD113-2	lightcyan
MIR376B	lightcyan
GPR132	lightcyan
NIPA1	lightcyan
GOLGA6L17P	lightcyan
LOC727924	lightcyan
TRPM1	lightcyan
DPH6	lightcyan
SEMA6D	lightcyan
FBN1	lightcyan
CYP19A1	lightcyan
ALDH1A2	lightcyan
LIPC	lightcyan
LOC102723344	lightcyan
APH1B	lightcyan
DPP8	lightcyan
PIAS1	lightcyan
GLCE	lightcyan
TUBGCP5	lightcyan
NDN	lightcyan
PWRN1	lightcyan
SNORD116-10	lightcyan
GOLGA8M	lightcyan
LOC105370757	lightcyan
PGBD4	lightcyan
EMC4	lightcyan
NOP10	lightcyan
TMCO5A	lightcyan
FAM98B	lightcyan
KNSTRN	lightcyan
MRPL42P5	lightcyan
EXD1	lightcyan
SLC24A5	lightcyan
EID1	lightcyan
COPS2	lightcyan

MAPK6	lightcyan
ARPP19	lightcyan
RSL24D1	lightcyan
MIR628	lightcyan
LIPC-AS1	lightcyan
RAB8B	lightcyan
CA12	lightcyan
RAB11A	lightcyan
ZWILCH	lightcyan
SNORD18B	lightcyan
FEM1B	lightcyan
SPESP1	lightcyan
LARP6	lightcyan
THAP10	lightcyan
MIR630	lightcyan
IMP3	lightcyan
RCN2	lightcyan
PSMA4	lightcyan
FAM103A1	lightcyan
TM6SF1	lightcyan
LOC101926911	lightcyan
LINC00924	lightcyan
SNRPA1	lightcyan
TM2D3	lightcyan
LPCAT2	lightcyan
FAM157C	lightcyan
NOMO3	lightcyan
NDUFAB1	lightcyan
ITFG1	lightcyan
TOX3	lightcyan
LINC00922	lightcyan
CES2	lightcyan
ADAMTS18	lightcyan
MEIOB	lightcyan
SNORA64	lightcyan
SNHG9	lightcyan
PRSS41	lightcyan
ERI2	lightcyan
LOC100190986	lightcyan
LOC101927814	lightcyan
CHP2	lightcyan
NPIPB9	lightcyan
SEPHS2	lightcyan
SNORA30	lightcyan
AHSP	lightcyan
ZNF267	lightcyan

VPS35	lightcyan
CES1	lightcyan
NUDT21	lightcyan
MT1X	lightcyan
ADGRG5	lightcyan
CFAP20	lightcyan
GOT2	lightcyan
RFWD3	lightcyan
TERF2IP	lightcyan
NUDT7	lightcyan
MAFTRR	lightcyan
DYNLRB2	lightcyan
MIR4720	lightcyan
HSBP1	lightcyan
OSGIN1	lightcyan
MIR6774	lightcyan
LYRM9	lightcyan
GAS7	lightcyan
PIGL	lightcyan
COPS3	lightcyan
CCDC144B	lightcyan
OMG	lightcyan
CDC27	lightcyan
ITGB3	lightcyan
LOC101927755	lightcyan
APPBP2	lightcyan
USP32	lightcyan
PRKAR1A	lightcyan
MAP2K6	lightcyan
SLC25A19	lightcyan
GLOD4	lightcyan
PITPNA-AS1	lightcyan
PAFAH1B1	lightcyan
OR3A2	lightcyan
OR3A1	lightcyan
LOC101928000	lightcyan
MIS12	lightcyan
TMEM256	lightcyan
MYH1	lightcyan
ADPRM	lightcyan
MAGOH2P	lightcyan
ZNF624	lightcyan
TRIM16L	lightcyan
ALDH3A1	lightcyan
SNORD42B	lightcyan
CPD	lightcyan

CCL2	lightcyan
CCL3	lightcyan
PIGW	lightcyan
PSMB3	lightcyan
SNORA21	lightcyan
LINC00672	lightcyan
LRRC3C	lightcyan
KRT39	lightcyan
KRT13	lightcyan
ACLY	lightcyan
C17orf105	lightcyan
MAPK8IP1P2	lightcyan
LOC102724532	lightcyan
GNGT2	lightcyan
LOC101927207	lightcyan
SLC35B1	lightcyan
MRPS23	lightcyan
CLTC	lightcyan
LOC653653	lightcyan
MIR4737	lightcyan
PSMD12	lightcyan
KPNA2	lightcyan
SLC16A6	lightcyan
LOC102723517	lightcyan
ATP5H	lightcyan
SUMO2	lightcyan
MIR4738	lightcyan
H3F3B	lightcyan
LOC105274304	lightcyan
ACTG1	lightcyan
IMPACT	lightcyan
WDR7	lightcyan
MYL12B	lightcyan
SEH1L	lightcyan
RNMT	lightcyan
OSBPL1A	lightcyan
MAPRE2	lightcyan
IER3IP1	lightcyan
VPS4B	lightcyan
USP14	lightcyan
CLUL1	lightcyan
CBX3P2	lightcyan
NDUFV2	lightcyan
NAPG	lightcyan
AFG3L2	lightcyan
CTAGE1	lightcyan

DSC2	lightcyan
DSG1	lightcyan
STARD6	lightcyan
LINC01416	lightcyan
LINC01539	lightcyan
NARS	lightcyan
TMX3	lightcyan
GTSCR1	lightcyan
FBXO15	lightcyan
CNDP2	lightcyan
LINC00909	lightcyan
ZNF43	lightcyan
JAK3	lightcyan
ZNF208	lightcyan
LINC00904	lightcyan
CEACAM21	lightcyan
ZNF223	lightcyan
ZIM2-AS1	lightcyan
ZNF556	lightcyan
MRPL54	lightcyan
TNFSF9	lightcyan
TUBB4A	lightcyan
LOC101928238	lightcyan
ICAM1	lightcyan
ZNF441	lightcyan
ZNF136	lightcyan
ZNF625-ZNF20	lightcyan
ZNF442	lightcyan
RNASEH2A	lightcyan
CLEC17A	lightcyan
MPV17L2	lightcyan
ZNF253	lightcyan
ZNF93	lightcyan
ZNF826P	lightcyan
ZNF85	lightcyan
LINC00664	lightcyan
ZNF257	lightcyan
ZNF676	lightcyan
ZNF729	lightcyan
ZNF728	lightcyan
ZNF730	lightcyan
ZNF681	lightcyan
UQCRFS1	lightcyan
HAMP	lightcyan
APLP1	lightcyan
TBCB	lightcyan

CAPNS1	lightcyan
PSMD8	lightcyan
LGALS16	lightcyan
PSMC4	lightcyan
LOC101928063	lightcyan
KLK8	lightcyan
SIGLEC12	lightcyan
MIR643	lightcyan
ZNF888	lightcyan
VN1R2	lightcyan
LILRA6	lightcyan
LAIR2	lightcyan
ZIM2	lightcyan
LOC105372476	lightcyan
DSTN	lightcyan
DTD1	lightcyan
FRG1BP	lightcyan
ITCH	lightcyan
PIGU	lightcyan
CSE1L	lightcyan
RAB22A	lightcyan
SNRPB	lightcyan
SNORA51	lightcyan
PRND	lightcyan
PCNA	lightcyan
TMEM230	lightcyan
GPCPD1	lightcyan
TRMT6	lightcyan
MCM8-AS1	lightcyan
BTBD3	lightcyan
NDUFAF5	lightcyan
SNRPB2	lightcyan
PET117	lightcyan
POLR3F	lightcyan
SEC23B	lightcyan
LINC00493	lightcyan
LOC100270804	lightcyan
NAA20	lightcyan
NKX2-4	lightcyan
APMAP	lightcyan
MLLT10P1	lightcyan
GSS	lightcyan
TRPC4AP	lightcyan
GHRH	lightcyan
SNHG17	lightcyan
MAFB	lightcyan

SERINC3	lightcyan
LINC01260	lightcyan
PIGT	lightcyan
SNORD12B	lightcyan
DPM1	lightcyan
PFDN4	lightcyan
AURKA	lightcyan
ATP5E	lightcyan
APP	lightcyan
USP16	lightcyan
RBM11	lightcyan
MIR548XHG	lightcyan
NCAM2	lightcyan
WRB	lightcyan
FAM3B	lightcyan
ABCC13	lightcyan
HSPA13	lightcyan
MIR125B2	lightcyan
CXADR	lightcyan
LINC00515	lightcyan
ATP5J	lightcyan
LINC00161	lightcyan
RWDD2B	lightcyan
LTN1	lightcyan
CCT8	lightcyan
GRIK1-AS2	lightcyan
URB1-AS1	lightcyan
PAXBP1-AS1	lightcyan
ATP5O	lightcyan
LINC01426	lightcyan
CBR3-AS1	lightcyan
PIGP	lightcyan
SH3BGR	lightcyan
MX1	lightcyan
U2AF1L5	lightcyan
LOC101928796	lightcyan
PIWIL3	lightcyan
ATP6V1E1	lightcyan
GSTT1	lightcyan
DMC1	lightcyan
KLHDC7B	lightcyan
SDF2L1	lightcyan
LOC391322	lightcyan
GSTT1-AS1	lightcyan
OSM	lightcyan
LIF	lightcyan

MIR659	lightcyan
APOBEC3B	lightcyan
SLC25A17	lightcyan
RBX1	lightcyan
XRCC6	lightcyan
PNPLA3	lightcyan
ACR	lightcyan
TMEM269	lightyellow
LINC01761	lightyellow
LEMD1	lightyellow
CR1L	lightyellow
NLRP3	lightyellow
HMGCL	lightyellow
SFN	lightyellow
HCRTR1	lightyellow
SPOCD1	lightyellow
MIR3605	lightyellow
C1orf94	lightyellow
LOC653160	lightyellow
LOC105378683	lightyellow
LOC339539	lightyellow
KLF17	lightyellow
FAAH	lightyellow
LEXM	lightyellow
LINC01359	lightyellow
LOC729930	lightyellow
CHI3L2	lightyellow
S100A3	lightyellow
S100A16	lightyellow
NTRK1	lightyellow
IGSF8	lightyellow
GAS5-AS1	lightyellow
LOC102724601	lightyellow
C4BPB	lightyellow
LINC00467	lightyellow
RD3	lightyellow
FLVCR1-AS1	lightyellow
PYCR2	lightyellow
AGT	lightyellow
LINC01132	lightyellow
LOC101929452	lightyellow
DGUOK-AS1	lightyellow
HJURP	lightyellow
LOC101928222	lightyellow
RHOB	lightyellow
SNORD53	lightyellow

LOC101929723	lightyellow
C2orf91	lightyellow
EPCAM	lightyellow
PARTICL	lightyellow
NCAPH	lightyellow
FAHD2B	lightyellow
SCTR	lightyellow
LOC105373764	lightyellow
LOC101927865	lightyellow
FAM124B	lightyellow
FLJ43879	lightyellow
EFHB	lightyellow
LINC00578	lightyellow
SH3BP5	lightyellow
SCN11A	lightyellow
LOC100132146	lightyellow
ADAMTS9	lightyellow
LOC105374313	lightyellow
LOC101928882	lightyellow
LIMD1-AS1	lightyellow
CISH	lightyellow
SNORD19B	lightyellow
ADAMTS9-AS1	lightyellow
MCM2	lightyellow
RBP2	lightyellow
LINC00881	lightyellow
LOC647323	lightyellow
LINC01091	lightyellow
GBA3	lightyellow
IGFBP7-AS1	lightyellow
ENPEP	lightyellow
NOP14-AS1	lightyellow
MIR548I2	lightyellow
FGFBP2	lightyellow
LINC01258	lightyellow
USP46-AS1	lightyellow
SPINK2	lightyellow
ALB	lightyellow
CXCL2	lightyellow
SFRP2	lightyellow
TRIM61	lightyellow
LOC100506085	lightyellow
LIFR-AS1	lightyellow
IL31RA	lightyellow
DDX4	lightyellow
ECSCR	lightyellow

LINC01265	lightyellow
FBXO4	lightyellow
FST	lightyellow
CRHBP	lightyellow
LINC01337	lightyellow
FAM170A	lightyellow
SLC25A2	lightyellow
SLC36A2	lightyellow
LOC100288254	lightyellow
NKX2-5	lightyellow
PEX7	lightyellow
EDN1	lightyellow
HLA-A	lightyellow
RNF5P1	lightyellow
HSD17B8	lightyellow
MIR5004	lightyellow
LCAL1	lightyellow
MTFR2	lightyellow
OSTCP1	lightyellow
MIR3939	lightyellow
LOC401286	lightyellow
LOC285889	lightyellow
FSCN1	lightyellow
SVOPL	lightyellow
WEE2	lightyellow
IL6	lightyellow
RPS2P32	lightyellow
SNHG15	lightyellow
FKBP9P1	lightyellow
SUMF2	lightyellow
KPNA7	lightyellow
STAG3L5P	lightyellow
POLR2J2	lightyellow
AKR1B15	lightyellow
LINC01003	lightyellow
RP1L1	lightyellow
FAM66B	lightyellow
XKR5	lightyellow
AZIN1-AS1	lightyellow
ZNF596	lightyellow
LINC00965	lightyellow
USP17L1	lightyellow
LOC392196	lightyellow
USP17L2	lightyellow
NUDT18	lightyellow
STMN4	lightyellow

C8orf4	lightyellow
PLAT	lightyellow
RP1	lightyellow
OSR2	lightyellow
NUDT2	lightyellow
LOC158435	lightyellow
LOC100499484-C9ORF174	lightyellow
TMEFF1	lightyellow
PALM2-AKAP2	lightyellow
XLOC_007697	lightyellow
LOC403323	lightyellow
LOC102725126	lightyellow
RPSAP9	lightyellow
CTSL	lightyellow
LOC642943	lightyellow
FOXE1	lightyellow
PSMD5-AS1	lightyellow
OR1L1	lightyellow
OR1K1	lightyellow
MIR6855	lightyellow
MIR4674	lightyellow
MAN1B1-AS1	lightyellow
RBM3	lightyellow
PFKFB1	lightyellow
AWAT1	lightyellow
KCNE5	lightyellow
DCAF12L1	lightyellow
FAM58A	lightyellow
LOC105373383	lightyellow
HECTD2-AS1	lightyellow
CYP17A1	lightyellow
C10orf67	lightyellow
SVILP1	lightyellow
FAM21EP	lightyellow
SGMS1-AS1	lightyellow
TMEM254-AS1	lightyellow
DYDC1	lightyellow
CEP55	lightyellow
PDLIM1	lightyellow
VAX1	lightyellow
PRAP1	lightyellow
FRG2B	lightyellow
NECTIN1	lightyellow
MUC5AC	lightyellow
TSSC2	lightyellow
TM7SF2	lightyellow

SLC25A45	lightyellow
TOLLIP-AS1	lightyellow
LINC01219	lightyellow
H19	lightyellow
HOTS	lightyellow
OR7E12P	lightyellow
RBMXL2	lightyellow
ADM	lightyellow
MTRNR2L8	lightyellow
MRGPRX3	lightyellow
SAA1	lightyellow
SAA2	lightyellow
OR10Q1	lightyellow
TKFC	lightyellow
SCGB1D2	lightyellow
FAM86C1	lightyellow
MIR3165	lightyellow
DEUP1	lightyellow
LOC387810	lightyellow
HMBS	lightyellow
LOC100507431	lightyellow
VWF	lightyellow
NUP37	lightyellow
SPSB2	lightyellow
ZNF705A	lightyellow
C12orf71	lightyellow
MIR7851	lightyellow
MIR4698	lightyellow
H1FNT	lightyellow
MIR4701	lightyellow
C1QL4	lightyellow
GPD1	lightyellow
INHBC	lightyellow
LOC101927901	lightyellow
IL22	lightyellow
LOC101928617	lightyellow
ALKBH2	lightyellow
RASAL1	lightyellow
COL4A1	lightyellow
GJA3	lightyellow
SNORD102	lightyellow
LINC01080	lightyellow
LINC01054	lightyellow
RNF212B	lightyellow
SFTA3	lightyellow
ESRRB	lightyellow

KCNK10	lightyellow
RNASE7	lightyellow
CEBPE	lightyellow
GZMB	lightyellow
NKX2-1-AS1	lightyellow
NKX2-8	lightyellow
FOS	lightyellow
BDKRB1	lightyellow
SEMA7A	lightyellow
GOLGA8IP	lightyellow
PWRN3	lightyellow
LOC101928227	lightyellow
LOC101928414	lightyellow
SLC28A2	lightyellow
C2CD4B	lightyellow
PPIB	lightyellow
LOC338963	lightyellow
BNC1	lightyellow
IDH2	lightyellow
LINC01582	lightyellow
ABCC11	lightyellow
LOC105371267	lightyellow
PRSS27	lightyellow
NMRAL1	lightyellow
ACSM3	lightyellow
SHCBP1	lightyellow
SYCE1L	lightyellow
MIR6504	lightyellow
TRAPPC2L	lightyellow
PRR11	lightyellow
OR1A2	lightyellow
GLTPD2	lightyellow
CCT6B	lightyellow
SLFN5	lightyellow
CSF3	lightyellow
TOP2A	lightyellow
KRT35	lightyellow
DHX58	lightyellow
TMEM92	lightyellow
RNF126P1	lightyellow
LOC102723505	lightyellow
LINC01152	lightyellow
CPSF4L	lightyellow
LOC100132174	lightyellow
LOC101928674	lightyellow
SOCS3	lightyellow

CBX4	lightyellow
MIR6787	lightyellow
FN3K	lightyellow
LINC00526	lightyellow
LIPG	lightyellow
SERPINB8	lightyellow
LINC00305	lightyellow
MIDN	lightyellow
FBN3	lightyellow
ILF3-AS1	lightyellow
FAAP24	lightyellow
WDR88	lightyellow
KIRREL2	lightyellow
SDHAF1	lightyellow
LOC100631378	lightyellow
ZFP36	lightyellow
LGALS17A	lightyellow
CYP2A13	lightyellow
PSG1	lightyellow
PSG7	lightyellow
RELB	lightyellow
TRAPPC6A	lightyellow
C19orf48	lightyellow
ETFB	lightyellow
LENG9	lightyellow
AURKC	lightyellow
LINC00851	lightyellow
CD93	lightyellow
FAM182A	lightyellow
ARHGAP40	lightyellow
LOC100128988	lightyellow
FAM209A	lightyellow
BTG3	lightyellow
KRTAP11-1	lightyellow
TFF3	lightyellow
PICSAR	lightyellow
LINC01310	lightyellow
MN1	lightyellow
CCT8L2	lightyellow
PIK3IP1	lightyellow
IL2RB	lightyellow
APOBEC3H	lightyellow
FRG1CP	lightyellow
TRABD2B	green
NBPF8	green
NBPF25P	green

NBPF10	green
TTC34	green
TNFRSF9	green
CASZ1	green
C1orf127	green
TMEM51-AS1	green
ESPNP	green
MFAP2	green
LDLRAD2	green
IFNLR1	green
RCC1	green
LOC101929464	green
CSMD2-AS1	green
TFAP2E	green
ERI3-IT1	green
KDM4A-AS1	green
ACOT11	green
MROH7-TTC4	green
LOC101926964	green
MSH4	green
SRGAP2-AS1	green
RORC	green
NUP210L	green
SPTA1	green
HMCN1	green
KIF14	green
IRF6	green
LOC101930114	green
GCSAML	green
DDX11L1	green
WASH7P	green
LOC100133331	green
LOC100288069	green
FAM87B	green
PLEKHN1	green
KLHL17	green
RNF223	green
TAS1R3	green
SCNN1D	green
ATAD3C	green
MMP23B	green
CALML6	green
LOC115110	green
LOC100996583	green
MIR4251	green
ARHGEF16	green

LINC01134	green
MIR4689	green
UTS2	green
LOC102724552	green
LINC01759	green
PIK3CD-AS1	green
PIK3CD-AS2	green
MIR5697	green
MTOR-AS1	green
NPPA-AS1	green
NPPB	green
TMEM51	green
C1orf195	green
CELA2A	green
CLCNKB	green
CLCNKA	green
HSPB7	green
EPHA2	green
ARHGEF19	green
SPATA21	green
CROCCP3	green
CROCCP2	green
CROCC	green
AKR7L	green
CDA	green
LOC100506801	green
CNR2	green
IL22RA1	green
LOC284632	green
RUNX3	green
RHD	green
MIR3917	green
CNKSR1	green
FAM110D	green
FCN3	green
FGR	green
SNORA44	green
RAB42	green
RNU11	green
SNORD103C	green
SERINC2	green
TINAGL1	green
MIR5585	green
LCK	green
DCDC2B	green
FAM167B	green

TSSK3	green
FAM229A	green
HMGB4	green
GJA4	green
TEKT2	green
MIR4255	green
ZC3H12A	green
MIR6732	green
GJA9-MYCBP	green
LOC105378663	green
TMCO2	green
MIR30E	green
SLFNL1-AS1	green
SLFNL1	green
CLDN19	green
LOC101929592	green
ARTN	green
MIR6079	green
SNORA110	green
C1orf228	green
SNORD38B	green
TSPAN1	green
RAD54L	green
FAAHP1	green
LINC01398	green
DMBX1	green
PDZK1IP1	green
LINC01389	green
FOXD2-AS1	green
FOXD2	green
TXNDC12-AS1	green
ECHDC2	green
MIR5095	green
MIR1273F	green
PODN	green
SLC1A7	green
DIO1	green
SSBP3-AS1	green
FAM151A	green
MROH7	green
TTC4	green
MGC34796	green
UBE2U	green
NEXN-AS1	green
UOX	green
BARHL2	green

LOC100129046	green
EPS8L3	green
LINC01397	green
LINC01160	green
MIR4256	green
FAM19A3	green
LOC100996251	green
LINC01762	green
LOC102723769	green
PFN1P2	green
ITGA10	green
MIR6736	green
HIST2H2BC	green
MTMR11	green
ADAMTSL4-AS1	green
BNIPL	green
TMOD4	green
LINGO4	green
S100A4	green
S100A14	green
NPR1	green
LOC343052	green
C1orf189	green
TDRD10	green
UBE2Q1-AS1	green
LENEP	green
EFNA4	green
MUC1	green
POU5F1P4	green
TSACC	green
VHLL	green
MIR9-1	green
PEAR1	green
INSRR	green
MIR765	green
ACKR1	green
FCER1A	green
VSIG8	green
ATP1A4	green
LOC729867	green
LY9	green
SLAMF7	green
FCGR3B	green
C1orf111	green
C1orf226	green
CCDC190	green

MIR1255B2	green
LOC100505918	green
LOC101928650	green
FMO1	green
FASLG	green
SERPINC1	green
LOC101928696	green
LINC01657	green
MIR488	green
LHX4-AS1	green
TEDDM1	green
PRG4	green
CFH	green
C1orf106	green
MYOG	green
MYBPH	green
SNORA77	green
LINC00260	green
LOC101929441	green
MIR135B	green
PM20D1	green
PIGR	green
LOC148696	green
CD34	green
HHIPL2	green
LOC101927143	green
LOC101927164	green
MIR6741	green
HIST3H3	green
LOC149373	green
MT1HL1	green
LINC01139	green
MIR3123	green
C1orf100	green
LINC01743	green
LINC01341	green
MIR3916	green
GCSAML-AS1	green
OR2C3	green
OR11L1	green
ZNF692	green
RGPD2	green
MLK7-AS1	green
PXDN	green
COLEC11	green
DCDC2C	green

MFSD2B	green
FAM179A	green
PLEKHH2	green
C2orf61	green
PROM2	green
ITPRIPL1	green
IL1R2	green
MIR548AU	green
SULT1C2	green
TFCP2L1	green
LOC150776	green
NEB	green
LOC101929378	green
LOC101927156	green
FZD7	green
PAX3	green
MLPH	green
GPR35	green
SNED1	green
LINC01247	green
LINC00487	green
LOC101929733	green
LINC01804	green
NT5C1B	green
MIR1301	green
GAREM2	green
CENPA	green
ABHD1	green
KRTCAP3	green
FLJ31356	green
LOC105374389	green
LOC388942	green
OXER1	green
HAAO	green
SIX3	green
SIX2	green
HCG2040054	green
LHCGR	green
MIR5192	green
LINC00309	green
MEIS1-AS3	green
LINC01797	green
SNORA36C	green
ANKRD53	green
NOTO	green
ACTG2	green

LBX2-AS1	green
LOC102724579	green
SFTPB	green
MIR6071	green
ANAPC1P1	green
CD8B	green
TEX37	green
LYG2	green
LYG1	green
LINC01104	green
LINC01796	green
MIR4265	green
FOXD4L1	green
FAM138B	green
LOC100506797	green
MIR4783	green
PROC	green
MIR4784	green
C2orf27B	green
DARS-AS1	green
LOC105373656	green
LOC101929260	green
MIR1258	green
FRZB	green
COL3A1	green
SDPR	green
LOC101927795	green
MIR7845	green
LOC105373878	green
RUFY4	green
CXCR2P1	green
CXCR2	green
MIR6513	green
CATIP	green
VIL1	green
WNT6	green
PRKAG3	green
IHH	green
SLC23A3	green
MIR3132	green
ASIC4	green
KCNE4	green
C2orf83	green
AGAP1-IT1	green
GBX2	green
KLHL30	green

ESPNL	green
SCLY	green
MIR4440	green
LOC200772	green
PDCD1	green
CCDC36	green
VGLL3	green
MECOM	green
CAV3	green
DLEC1	green
PTH1R	green
CCR2	green
COL7A1	green
MST1R	green
COL8A1	green
IL20RB	green
SPSB4	green
LOC440982	green
WWTR1	green
IGF2BP2	green
GMNC	green
ATP13A4	green
SDHAP1	green
CIDEC	green
GHRL	green
GHRLOS	green
HDAC11-AS1	green
MIR563	green
LINC00691	green
MIR548AY	green
MIR6822	green
CCR8	green
LOC729083	green
LARS2-AS1	green
TDGF1	green
NBEAL2	green
TMEM89	green
CELSR3-AS1	green
MIR4793	green
C3orf84	green
AMIGO3	green
MST1	green
MIR5193	green
GNAT1	green
MIR5787	green
ZMYND10	green

ACY1	green
IQCF1	green
LINC00696	green
TLR9	green
TNNC1	green
SNORD136	green
MIR8064	green
ITIH3	green
ITIH4-AS1	green
ITIH4	green
FLNB-AS1	green
MAGI1-AS1	green
FOXP1-AS1	green
TMEM30CP	green
PLCXD2-AS1	green
MIR8076	green
ARHGAP31-AS1	green
PLA1A	green
MIR7110	green
MYLK-AS2	green
MIR548I1	green
ALDH1L1-AS1	green
CFAP100	green
TXNRD3NB	green
MIR7976	green
GATA2	green
GATA2-AS1	green
RAB43	green
COL6A4P2	green
CLDN18	green
DZIP1L	green
FOXL2	green
FOXL2NB	green
LOC101927866	green
ZIC4	green
ZIC1	green
AGTR1	green
WWTR1-AS1	green
LOC105374205	green
ABCC5-AS1	green
HTR3C	green
HTR3E-AS1	green
HTR3E	green
MIR1224	green
SNORD66	green
IGF2BP2-AS1	green

NMRAL2P	green
ADIPOQ	green
FLJ42393	green
OSTN-AS1	green
XXYLT1-AS1	green
XXYLT1-AS2	green
LOC105374297	green
MUC20	green
MIR6829	green
TM4SF19-TCTEX1D2	green
UBXN7-AS1	green
LEF1	green
IL15	green
LOC101930370	green
EVC2	green
CPEB2-AS1	green
CNGA1	green
FTLP10	green
ADH6	green
TET2-AS1	green
SLC7A11-AS1	green
ANXA10	green
HAND2-AS1	green
KLKB1	green
LOC339975	green
LOC100129917	green
SLC26A1	green
IDUA	green
CTBP1-AS	green
TACC3	green
HTT-AS	green
HGFAC	green
S100P	green
LOC100129931	green
LOC389199	green
USP17L10	green
SLC34A2	green
LOC401127	green
LVCAT1	green
YIPF7	green
ZAR1	green
MIR4449	green
UGT2B17	green
PF4V1	green
PF4	green
PPBP	green

NKX6-1	green
ADH1A	green
LEF1-AS1	green
MIR302C	green
CCNA2	green
PABPC4L	green
LINC00616	green
MGARP	green
UCP1	green
LOC100507639	green
SMAD1-AS2	green
LOC729870	green
LOC101928052	green
TKTL2	green
LINC01179	green
LOC101928131	green
HAND2	green
HPGD	green
WWC2-AS2	green
ANKRD37	green
EBF1	green
BTNL8	green
C5orf38	green
MROH2B	green
OCLN	green
LINC00491	green
LINC00992	green
LOC101927421	green
TH2LCRR	green
ANKHD1-EIF4EBP3	green
PCDHGA1	green
IL17B	green
GLRA1	green
ADAMTS2	green
HRAT5	green
LOC100996325	green
ZDHHC11	green
MIR4635	green
SLC12A7	green
CTD-3080P12.3	green
MIR6075	green
IRX2	green
IRX1	green
MIR6131	green
MIR4279	green
C7	green

MIR4459	green
GZMK	green
LOC441081	green
LOC102477328	green
FOXD1	green
NCRUPAR	green
CTD-2201I18.1	green
LOC102546226	green
HNCAT21	green
MGC32805	green
TEX43	green
LOC105379176	green
LEAP2	green
LOC105379183	green
TCF7	green
LOC102546229	green
PITX1	green
C5orf66-AS1	green
C5orf66-AS2	green
VTRNA2-1	green
SMAD5-AS1	green
MIR874	green
GFRA3	green
KIF20A	green
SNORD63	green
SLC23A1	green
MZB1	green
EIF4EBP3	green
VTRNA1-1	green
VTRNA1-2	green
PCDHGA8	green
PCDHGA12	green
PCDHGB8P	green
SPRY4	green
SPRY4-IT1	green
PLAC8L1	green
CARMN	green
MIR145	green
MIR378A	green
FAT2	green
CTB-113P19.1	green
MIR1303	green
LOC101927740	green
CCNJL	green
MIR218-2	green
LOC105377716	green

GABRP	green
TLX3	green
EFCAB9	green
HK3	green
FGFR4	green
DOK3	green
PRR7-AS1	green
LOC101928445	green
LOC105377763	green
SNORD95	green
BMP6	green
GMPR	green
LOC285819	green
MDGA1	green
KCNK5	green
C6orf132	green
CYP39A1	green
TBX18	green
GABRR1	green
ADGRG6	green
RAET1E	green
LOC102723831	green
SMOC2	green
HUS1B	green
FOXCUT	green
LINC01600	green
MIR3691	green
LOC101927972	green
TFAP2A-AS1	green
MAK	green
LOC100130357	green
LINC01108	green
JARID2-AS1	green
LOC105374952	green
RNF144B	green
SOX4	green
C6orf229	green
SLC17A4	green
SLC17A3	green
HIST1H3C	green
BTN2A3P	green
OR2B6	green
HCG14	green
OR2H1	green
OR2H2	green
SNORD32B	green

HLA-F-AS1	green
IFITM4P	green
HCG9	green
HLA-J	green
MIR6891	green
LINC00243	green
PSORS1C2	green
POU5F1	green
PSORS1C3	green
HCG27	green
MICA	green
LINC01149	green
DDX39B	green
LTA	green
MCCD1	green
DDX39B-AS1	green
MIR4646	green
LY6G6E	green
LY6G5C	green
LY6G6F	green
LY6G6C	green
C6orf25	green
SLC44A4	green
CYP21A1P	green
CYP21A2	green
MIR6721	green
PPT2-EGFL8	green
MIR3934	green
MIR6835	green
SCUBE3	green
TULP1	green
TEAD3	green
LOC285847	green
ARMC12	green
PRICKLE4	green
PTCRA	green
CRIP3	green
LOC101926934	green
LOC101926962	green
TFAP2B	green
LGSN	green
MIR30C2	green
MIR30A	green
LINC00472	green
KHDC1L	green
FAM46A	green

GABRR2	green
FHL5	green
FAM26E	green
BRD7P3	green
VNN1	green
VNN3	green
SNORD100	green
MIR3145	green
PHACTR2-AS1	green
SNORA98	green
LOC105378047	green
MYCT1	green
NOX3	green
MIR3692	green
EZR-AS1	green
LPAL2	green
C6orf118	green
LINC01558	green
KIF25-AS1	green
FRMD1	green
LINC01615	green
LVCAT5	green
CPA1	green
GRID2IP	green
LOC100505921	green
ABCB5	green
DNAH11	green
TBX20	green
TRG-AS1	green
NPC1L1	green
C7orf69	green
TNS3	green
ELDR	green
LOC650226	green
GTF2IP1	green
GATSL2	green
SPDYE8P	green
UPK3B	green
RASA4	green
LOC101927870	green
LAMB4	green
FAM71F2	green
SMO	green
SLC13A4	green
OR2A1-AS1	green
GBX1	green

LOC442497	green
LOC101926963	green
HRAT92	green
CYP2W1	green
UNCX	green
MICALL2	green
TMEM184A	green
ELFN1-AS1	green
MIR4655	green
MIR6836	green
LOC101927181	green
MIR4648	green
GRIFIN	green
MIR4656	green
PAPOLB	green
MIR589	green
RNF216-IT1	green
MIR6874	green
LOC101927354	green
LOC401312	green
SNORD93	green
C7orf71	green
TRIL	green
LOC101928168	green
MIR550A3	green
INMT	green
LINC01450	green
SPDYE1	green
AEBP1	green
MIR6837	green
LINC00525	green
EGFR-AS1	green
LOC101928401	green
LOC100996437	green
LINC01372	green
SPDYE7P	green
TRIM74	green
NSUN5P2	green
STAG3L1	green
MIR4284	green
CLDN3	green
ABHD11-AS1	green
TRIM73	green
SPDYE5	green
CCL26	green
MIR4651	green

FDPSP2	green
MAGI2-AS2	green
LOC102723885	green
LOC101409256	green
COL1A2	green
PON1	green
LOC100506136	green
BAIAP2L1	green
LOC101927550	green
AZGP1P1	green
MIR93	green
MIR106B	green
PILRA	green
STAG3L5P-PVRIG2P-PILRB	green
SPDYE3	green
C7orf61	green
PCOLCE	green
PCOLCE-AS1	green
MIR6875	green
MYL10	green
MIR4285	green
SPDYE6	green
LOC100289561	green
PRKRIP1	green
MIR4467	green
POLR2J3	green
SLC26A3	green
LSMEM1	green
ST7-OT4	green
FEZF1	green
FEZF1-AS1	green
LMOD2	green
FSCN3	green
LINC01000	green
OPN1SW	green
KCP	green
TSGA13	green
LOC100506937	green
MIR6133	green
PRSS37	green
TAS2R38	green
OR9A4	green
MGAM	green
MOXD2P	green
OR2F2	green
ARHGEF34P	green

LINC00996	green
AOC1	green
MIR3907	green
LOC101929998	green
MNX1	green
MIR595	green
LINC00689	green
TNFRSF10A	green
LOC729732	green
NUGGC	green
LOC101929450	green
MIR1268A	green
ADAM5	green
LOC101929415	green
PKHD1L1	green
LOC105375734	green
COL22A1	green
MROH5	green
SPATC1	green
KBTBD11-OT1	green
ANGPT2	green
MIR8055	green
MIR597	green
SLC35G5	green
DEFB135	green
SLC7A2	green
DOK2	green
BIN3-IT1	green
TNFRSF10D	green
LOC389641	green
MIR6843	green
RBPMS-AS1	green
HTRA4	green
DKK4	green
TRIM55	green
RRS1-AS1	green
PTTG3P	green
MCMDC2	green
PPP1R42	green
RDH10-AS1	green
STAU2-AS1	green
GEM	green
SNX31	green
LOC105375713	green
LOC101927543	green
LINC01151	green

PCAT1	green
PCAT2	green
ASAP1-IT2	green
TMEM71	green
LOC101927822	green
MIR30B	green
GPR20	green
SLURP1	green
C8orf31	green
MROH6	green
LOC101928160	green
BREA2	green
MIR661	green
WDR97	green
LOC101928902	green
TONSL-AS1	green
FOXH1	green
UNQ6494	green
LINC01492	green
MLANA	green
PGM5	green
GNA14-AS1	green
C9orf153	green
C9orf84	green
COL27A1	green
PKN3	green
C9orf50	green
CFAP77	green
PNPLA7	green
GLIS3-AS1	green
IFNA14	green
IZUMO3	green
LINC01251	green
CNTFR-AS1	green
CCL21	green
DNAJB5-AS1	green
C9orf131	green
SIT1	green
TPM2	green
LINC00961	green
LOC105379252	green
LOC102723709	green
FAM27E2	green
FAM27E3	green
ANKRD20A4	green
TMEM252	green

LINC01504	green
LINC01474	green
VPS13A-AS1	green
LOC101927623	green
LOC100129316	green
LINC00484	green
LINC00475	green
SNORA84	green
NUTM2F	green
TAL2	green
ACTL7A	green
LRRC37A5P	green
SLC46A2	green
ORM2	green
OR1J2	green
OR1J4	green
MIR601	green
MIR7150	green
MIR181A2	green
MIR181B2	green
WDR38	green
TTC16	green
CFAP157	green
ENG	green
SLC25A25-AS1	green
LOC100506100	green
PHYHD1	green
LOC401554	green
QRFP	green
GFI1B	green
ABO	green
STKLD1	green
LOC100130548	green
COL5A1-AS1	green
LOC101928525	green
LCN9	green
HSPC324	green
MIR126	green
LCN8	green
LCN6	green
LCN10	green
LOC100128593	green
CCDC183-AS1	green
MIR4292	green
C8G	green
MIR4479	green

MAMDC4	green
CLIC3	green
TMEM210	green
CYSRT1	green
FAM166A	green
ENTPD8	green
EHMT1-IT1	green
MIR602	green
LOC101928389	green
RBMXL3	green
KIAA1210	green
OR13H1	green
LOC340581	green
MXRA5	green
PRKX-AS1	green
VCX3B	green
TLR8-AS1	green
NHS-AS1	green
LOC729609	green
SNORA11C	green
UXT-AS1	green
ZNF630	green
ZNF630-AS1	green
FOXP3	green
PRICKLE3	green
GAGE10	green
MIR6894	green
MTRNR2L10	green
AWAT2	green
DGAT2L6	green
DLG3-AS1	green
ITGB1BP2	green
GCNA	green
RPS26P11	green
MIR545	green
ARL13A	green
XKRX	green
SLC25A53	green
TDGF1P3	green
SNORA35	green
MIR766	green
SOWAHD	green
LINC01402	green
NKAPP1	green
LOC101928402	green
MIR503HG	green

CT45A10	green
SAGE1	green
ZIC3	green
LOC645188	green
MAGEC1	green
CSAG4	green
CSAG1	green
TREX2	green
AVPR2	green
HCFC1-AS1	green
SLC16A12-AS1	green
SLC16A12	green
AKR1C3	green
BEND7	green
ACBD7-DCLRE1CP1	green
ST8SIA6-AS1	green
CUBN	green
LOC102031319	green
LOC105378269	green
COL13A1	green
CDH23-AS1	green
PLA2G12B	green
P4HA1	green
LOXL4	green
WNT8B	green
TUBAL3	green
GATA3	green
MIR548AK	green
MIR4481	green
OLAH	green
PPIAP30	green
MIR1915	green
EBLN1	green
LINC00264	green
MIR8086	green
LOC101929352	green
LINC00840	green
LOC102724323	green
MIR4294	green
MIR605	green
MIR548F1	green
SLC16A9	green
POU5F1P5	green
LOC101928994	green
NEUROG3	green
NODAL	green

UNC5B-AS1	green
OIT3	green
NUTM2B	green
LINC00858	green
MIR346	green
LIPN	green
IFIT1B	green
MARK2P9	green
PLCE1-AS2	green
PLCE1-AS1	green
MIR6507	green
MIR608	green
LBX1-AS1	green
LBX1	green
NFKB2	green
C10orf95	green
AS3MT	green
MIR4482	green
SORCS3-AS1	green
SNORA87	green
LOC102724589	green
MIR4681	green
MIR4682	green
LINC01561	green
ATE1-AS1	green
ARMS2	green
MIR3941	green
FAM24B-CUZD1	green
FAM24A	green
MIR4296	green
MMP21	green
FANK1-AS1	green
AS-PTPRE	green
ADAM8	green
VENTX	green
LOC283194	green
LINC01001	green
ART1	green
PLEKHA7	green
PAUPAR	green
DKFZp686K1684	green
TSPAN18	green
AHNAK	green
ACTN3	green
CCDC83	green
CCDC81	green

PGR	green
LOC101929011	green
SCGB1C1	green
ANO9	green
LMNTD2	green
DRD4	green
EPS8L2	green
CRACR2B	green
LOC101927503	green
MUC5B	green
KRTAP5-2	green
IGF2	green
IGF2-AS	green
MIR4686	green
TRPM5	green
CHRNA10	green
OR52B4	green
OR51E1	green
OLFM5P	green
OR52B6	green
OR56A4	green
LOC102724784	green
C11orf16	green
ASCL3	green
MYOD1	green
SPTY2D1-AS1	green
CSRP3	green
NAV2-AS4	green
MIR4486	green
MIR4694	green
NAV2-AS2	green
DBX1	green
MIR3973	green
LOC101928510	green
LARGE2	green
MIR4688	green
F2	green
LOC101928943	green
MYBPC3	green
MIR4487	green
RAPSN	green
FAM180B	green
SMTNL1	green
OR10V1	green
VWCE	green
PGA5	green

C11orf98	green
HRASLS2	green
PLCB3	green
CCDC88B	green
SLC22A12	green
CDC42BPG	green
GPHA2	green
MIR194-2HG	green
TMEM262	green
NAALADL1	green
SPDYC	green
SLC22A20	green
NEAT1	green
KCNK7	green
OVOL1	green
CATSPER1	green
LOC101928069	green
RBM14	green
C11orf86	green
GPR152	green
CABP4	green
DOC2GP	green
TBX10	green
MIR4691	green
MIR7113	green
MRGPRD	green
MRGPRF-AS1	green
MRGPRF	green
FGF3	green
MIR6754	green
IL18BP	green
FOLR1	green
PHOX2A	green
FOLR3	green
MIR4692	green
ATG16L2	green
P2RY2	green
UCP3	green
LOC101928580	green
P4HA3	green
OR2AT4	green
MIR326	green
OMP	green
LOC646029	green
KCTD14	green
THRSP	green

LOC101929295	green
LOC101054525	green
MMP10	green
CASP12	green
HSPB2	green
LOC105369509	green
APOA5	green
FXYD2	green
JAML	green
LOC100131626	green
CXCR5	green
PDZD3	green
LOC101929227	green
MIR100	green
LOC101929340	green
LOC101929427	green
MIR3167	green
LOC101929538	green
SENCR	green
NTM-IT	green
SLC38A4	green
MYRFL	green
ANO2	green
PLEKHG6	green
TMPRSS12	green
KRT18	green
MMP19	green
LRP1-AS	green
LOC101929058	green
STAB2	green
TRPV4	green
LHX5	green
TBX5	green
HPD	green
FAM138D	green
SLC6A12	green
LOC102723544	green
LINC00942	green
LOC283440	green
FOXM1	green
LOC101929549	green
NTF3	green
SCARNA11	green
ACRBP	green
LAG3	green
C1RL-AS1	green

POU5F1P3	green
FAM90A1	green
A2ML1	green
LINC00612	green
CLEC12A	green
CLEC1B	green
KLRD1	green
PRB4	green
RERGL	green
GYS2	green
REP15	green
DENND5B-AS1	green
LOC105369723	green
ENDOU	green
OR8S1	green
MIR1293	green
HIGD1C	green
KRT8	green
NPFF	green
NFE2	green
GDF11	green
PMEL	green
LOC105369781	green
SLC39A5	green
IL23A	green
GPR182	green
STAC3	green
GLI1	green
MIR6758	green
LOC101927583	green
CYP27B1	green
MIRLET7I	green
SNORA70G	green
LOC100130075	green
ALX1	green
LOC100507616	green
LOC102724933	green
MIR492	green
MIR331	green
CCDC38	green
HAL	green
GAS2L3	green
PMCH	green
MIR619	green
DAO	green
FOXN4	green

HVCN1	green
MIR6861	green
MIR6762	green
LHX5-AS1	green
TBX5-AS1	green
TBX3	green
LOC100506551	green
MIR7107	green
CLIP1-AS1	green
HCAR2	green
HCAR3	green
MIR4304	green
LOC101927415	green
FLJ37505	green
MIR3612	green
LACAT8	green
POLE	green
RNF17	green
RXFP2	green
RNF113B	green
SLC15A1	green
LOC101927437	green
BASP1P1	green
USP12-AS1	green
URAD	green
LINC00332	green
MIR8079	green
SNORA31	green
SLC25A30-AS1	green
LINC00282	green
LOC100288208	green
POU4F1	green
LINC01068	green
LOC101927248	green
SOX21-AS1	green
MIR3170	green
ZIC5	green
LINC00554	green
ZIC2	green
PROZ	green
F10	green
F10-AS1	green
GRTP1	green
GAS6-AS1	green
LINC00565	green
SLC10A1	green

RALGAPA1P1	green
LOC644919	green
LINC01588	green
SAMD4A	green
LOC101927690	green
SYNE2	green
LTBP2	green
TSHR	green
LOC101929718	green
TPPP2	green
RNASE13	green
PRMT5-AS1	green
AJUBA	green
LOC101926933	green
PSMB11	green
PABPN1	green
ZFHX2	green
FITM1	green
TGM1	green
NFATC4	green
ADCY4	green
CBLN3	green
GZMH	green
LOC102724890	green
MIR4307HG	green
SNORD127	green
POLE2	green
LINC01599	green
DLGAP5	green
OTX2	green
TOMM20L	green
SIX1	green
LOC101927780	green
HIF1A-AS2	green
GPHB5	green
LOC100506321	green
FAM71D	green
PAPLN	green
LOC101928123	green
PGF	green
NOXRED1	green
C14orf178	green
LOC101928909	green
ITPK1-AS1	green
IFI27	green
TCL6	green

MIR151B	green
MIR345	green
MIR300	green
DIO3OS	green
AMN	green
ZBTB42	green
TEX22	green
CRIP1	green
LOC105370697	green
CRTC3-AS1	green
HERC2P3	green
HERC2P2	green
ATP10A	green
LCTL	green
ITGA11	green
PAQR5	green
LINC00927	green
IL16	green
LOC400464	green
LINC01193	green
GOLGA6L2	green
GOLGA8K	green
NUTM1	green
LOC105370941	green
PHGR1	green
PPP1R14D	green
C15orf62	green
SPINT1	green
LOC105370943	green
DLL4	green
LOC105370792	green
PLA2G4B	green
SPTBN5	green
SERINC4	green
WDR76	green
HYPK	green
CEP152	green
LOC100422556	green
FOXB1	green
RBPMS2	green
KBTBD13	green
CILP	green
MIR4311	green
LOC101929151	green
THSD4-AS2	green
HEXA-AS1	green

LOC102723640	green
LOC729739	green
MIR4513	green
MIR6882	green
LMAN1L	green
MIR631	green
SNX33	green
LINC00597	green
LOC91450	green
SH2D7	green
CHRNA5	green
MTHFS	green
LOC642423	green
LINC00933	green
MIR1276	green
LINC00928	green
TICRR	green
MIR5009	green
ANPEP	green
TTLL13P	green
LINC00930	green
ASB9P1	green
MIR3175	green
IRAIN	green
PRKXP1	green
LOC102723335	green
SMG1P7	green
CLEC18B	green
PKD1P6-NPIPP1	green
MYH11	green
ABCC6	green
GPR139	green
NPIPB5	green
ERN2	green
SCNN1B	green
CLEC18A	green
LOC102724084	green
LOC100287036	green
CDH15	green
VPS9D1-AS1	green
PDIA2	green
LOC100134368	green
PRR35	green
MIR5587	green
MIR3176	green
WFIKKN1	green

LOC105371184	green
CCDC78	green
LOC105371038	green
PRR25	green
C1QTNF8	green
TPSG1	green
CCDC154	green
LOC105371046	green
EME2	green
MIR3177	green
SNORA78	green
RNF151	green
NOXO1	green
MIR3180-5	green
MIR4516	green
MIR1225	green
SNORD60	green
LOC105371049	green
LOC106660606	green
DNASE1L2	green
BRICD5	green
MIR3677	green
NTN3	green
MIR3178	green
MIR6768	green
PRSS30P	green
PRSS22	green
CLDN6	green
LINC00514	green
LOC101929613	green
CLDN9	green
IL32	green
MMP25	green
OR1F2P	green
ZNF205-AS1	green
LINC00921	green
C16orf90	green
NLRC3	green
SRL	green
PAM16	green
12.IX	green
C16orf71	green
EMP2	green
SOCS1	green
LOC105371083	green
MIR4718	green

LOC101927311	green
MIR193B	green
LOC105447648	green
PLA2G10	green
LOC100288162	green
LOC100505915	green
MIR3180-4	green
PKD1P1	green
ABCC6P1	green
ACSM1	green
ZP2	green
CRYM-AS1	green
NPIPB4	green
LOC283887	green
LCMT1-AS2	green
MIR548W	green
IL21R-AS1	green
NUPR1	green
SULT1A2	green
ATP2A1	green
LAT	green
C16orf92	green
GDPD3	green
MIR4519	green
HSD3B7	green
PRSS8	green
PRSS53	green
PYCARD-AS1	green
ITGAD	green
SLC5A2	green
LOC102723753	green
MIR3181	green
IRX5	green
IRX6	green
MT1DP	green
CCL22	green
CCDC102A	green
MIR6772	green
RRAD	green
ELMO3	green
HSF4	green
AGRP	green
ESRP2	green
TMED6	green
MIR1538	green
LOC400541	green

CLEC18C	green
LOC105371328	green
MTSS1L	green
VAC14-AS1	green
MARVELD3	green
PKD1L3	green
PMFBP1	green
HCCAT5	green
LOC105376772	green
CTRB2	green
LOC100506281	green
CTRB1	green
LINC01227	green
MIR7854	green
MIR8058	green
LOC101928417	green
LINC00311	green
MIR5093	green
LOC101928557	green
FENDRR	green
FOXC2	green
FOXL1	green
LOC440390	green
LOC101928708	green
LOC101928659	green
MIR6775	green
MIR5189	green
ZNF469	green
IL17C	green
LOC100289580	green
LOC339059	green
PABPN1L	green
LOC101927793	green
CDT1	green
LOC105371414	green
SLC22A31	green
SNORD68	green
MC1R	green
GAS8-AS1	green
URAHP	green
SENP3-EIF4A1	green
RAD51L3-RFFL	green
LOC102723471	green
LOC100131347	green
CRHR1-IT1-CRHR1	green
ARL17A	green

ABCC3	green
TBC1D3P1-DHX40P1	green
FAM20A	green
ST6GALNAC2	green
JMJD6	green
LOC101927727	green
MIR3183	green
TLCD2	green
SERPINF2	green
OVCA2	green
HIC1	green
LOC284009	green
MIR6776	green
LOC105371592	green
OR3A4P	green
TAX1BP3	green
ATP2A3	green
GP1BA	green
LOC102724009	green
MIR6865	green
MIR6864	green
FAM64A	green
XAF1	green
RNASEK-C17orf49	green
ALOX12	green
MIR195	green
CLEC10A	green
MIR324	green
TNK1	green
SPEM1	green
SLC35G6	green
TMEM88	green
MIR6883	green
MFSD6L	green
LINC00675	green
CDRT15P1	green
CDRT15	green
CDRT4	green
CDRT1	green
MIR1288	green
SNORD49B	green
FLCN	green
RAI1-AS1	green
SMCR5	green
MIR33B	green
MIR6777	green

FLJ35934	green
MIR1180	green
MFAP4	green
SLC47A2	green
CCDC144CP	green
LGALS9B	green
KCNJ18	green
PYY2	green
VTN	green
SEBOX	green
SPAG5-AS1	green
MIR4732	green
CRYBA1	green
DPRXP4	green
MIR193A	green
MIR365B	green
ARGFXP2	green
SLFN13	green
SLFN14	green
HEATR9	green
CCL5	green
MIR2909	green
C17orf78	green
TCAP	green
SNORD124	green
MIR6884	green
KRT20	green
KRT19	green
KRT15	green
GAST	green
ZNF385C	green
HSPB9	green
MIR5010	green
HSD17B1	green
AOC3	green
AOC2	green
MIR6781	green
ETV4	green
NAGS	green
C17orf53	green
ASB16	green
LINC01180	green
HIGD1B	green
GJC1	green
MAPT-AS1	green
MAPT-IT1	green

STH	green
KANSL1-AS1	green
LRRC37A2	green
MYL4	green
LRRC46	green
HOXB3	green
LOC105371814	green
B4GALNT2	green
NXPH3	green
TAC4	green
FLJ45513	green
HILS1	green
SGCA	green
COL1A1	green
ACSF2	green
MYCBPAP	green
LOC101927274	green
C17orf67	green
MTVR2	green
LOC101927557	green
LOC101927539	green
C17orf47	green
TBX2-AS1	green
C17orf82	green
TBX2	green
LOC101927855	green
CD79B	green
PRKCA-AS1	green
LOC101928021	green
LOC440461	green
ABCA9-AS1	green
LOC101928205	green
LOC146795	green
C17orf77	green
CD300LB	green
MIR6785	green
FOXJ1	green
ZACN	green
RNF157-AS1	green
QRICH2	green
SNORD1C	green
SNORD1B	green
LOC101928514	green
LOC105371907	green
C1QTNF1	green
C1QTNF1-AS1	green

ENGASE	green
CBX2	green
CCDC40	green
CARD14	green
MIR4730	green
LOC101928855	green
AATK-AS1	green
LOC105371925	green
MIR3186	green
MIR4740	green
FSCN2	green
PDE6G	green
TSPAN10	green
PPP1R27	green
LRRC45	green
LOC101929511	green
ZNF750	green
LOC100505549	green
NFATC1	green
MIR8078	green
TXNDC2	green
MIR4526	green
GATA6	green
SIGLEC15	green
ELOA2	green
SKOR2	green
MIR4743	green
MIR4744	green
SNORD58C	green
MIR4320	green
CFAP53	green
TCF4-AS1	green
LINC-ROR	green
PMAIP1	green
LINC01544	green
LOC101927481	green
LOC101060542	green
LINC00908	green
SALL3	green
CYP4F8	green
TLE2	green
CATSPERD	green
COL5A3	green
NWD1	green
WDR62	green
SIX5	green

SEC1P	green
NLRP4	green
WASH5P	green
ODF3L2	green
GZMM	green
CFD	green
EFNA2	green
CIRBP-AS1	green
ATP8B3	green
LOC100288123	green
CSNK1G2-AS1	green
MKNK2	green
TMPRSS9	green
MIR7850	green
TLE6	green
CACTIN-AS1	green
TJP3	green
APBA3	green
RAX2	green
MIR637	green
MIR4746	green
PLIN4	green
PLIN5	green
LOC100128568	green
LOC390877	green
PSPN	green
TNFSF14	green
TRIP10	green
SH2D3A	green
PCP2	green
FCER2	green
CD209	green
ZNF177	green
ZNF560	green
MIR638	green
ANGPTL8	green
KANK2	green
TSPAN16	green
RGL3	green
MIR7974	green
ZNF69	green
ZNF878	green
ZNF788	green
ZNF20	green
BEST2	green
DAND5	green

MIR24-2	green
LOC284454	green
MIR181C	green
RLN3	green
PODNL1	green
C19orf67	green
LOC100507373	green
NOTCH3	green
EPHX3	green
MIR1470	green
OR10H5	green
CALR3	green
F2RL3	green
USHBP1	green
ANKLE1	green
BST2	green
FAM129C	green
NXNL1	green
SNORA68	green
KIAA1683	green
MIR3188	green
MIR640	green
LOC400684	green
SLC7A9	green
LINC01801	green
FXYD3	green
UPK1A	green
ETV2	green
UPK1A-AS1	green
ZBTB32	green
LINC01529	green
NFKBID	green
LOC105372383	green
LOC101927572	green
SYNE4	green
OVOL3	green
LOC284412	green
RASGRP4	green
GGN	green
CAPN12	green
CCER2	green
NCCRP1	green
SELENOV	green
CNTD2	green
MIA	green
CYP2A6	green

CYP2A7	green
EGLN2	green
CYP2F1	green
ERICH4	green
PCAT19	green
CEACAM4	green
LOC100505622	green
CXCL17	green
CEACAM1	green
PINLYP	green
SRRM5	green
KCNN4	green
MIR4531	green
IGSF23	green
CEACAM16	green
APOC4-APOC2	green
EXOC3L2	green
CKM	green
DMPK	green
FOXA3	green
NANOS2	green
CCDC61	green
MIR769	green
LOC93429	green
HIF3A	green
CCDC8	green
MIR320E	green
NAPA-AS1	green
SNORD23	green
CCDC114	green
FAM83E	green
LOC105447645	green
NTN5	green
IZUMO1	green
FUT2	green
BCAT2	green
NUCB1-AS1	green
LOC101059948	green
LHB	green
NTF4	green
HRC	green
TEAD2	green
DKKL1	green
CCDC155	green
MIR150	green
RCN3	green

ADM5	green
TSKS	green
PTOV1-AS2	green
MIR4749	green
SIGLEC16	green
IZUMO2	green
ACPT	green
KLK14	green
SPACA6	green
SPACA6P-AS	green
MIR99B	green
ERVV-1	green
LOC284379	green
NCR1	green
EPS8L1	green
TMEM86B	green
COX6B2	green
TMEM238	green
SBK3	green
SBK2	green
ZSCAN4	green
ZNF552	green
RNF225	green
FAM65C	green
TMC2	green
SIGLEC1	green
NINL	green
SLA2	green
LOC100287792	green
LPIN3	green
MYBL2	green
NPEPL1	green
TCF15	green
SLC52A3	green
SDCBP2	green
GNRH2	green
UBOX5-AS1	green
ADAM33	green
LINC01730	green
MIR103A2	green
LINC01433	green
C20orf78	green
LOC100130264	green
KIZ-AS1	green
CST1	green
CST7	green

LOC101926935	green
LOC100134868	green
MIR663AHG	green
REM1	green
LINC00028	green
HM13-AS1	green
COX4I2	green
FOXS1	green
LOC101929698	green
C20orf144	green
ACTL10	green
ZNF341-AS1	green
ASIP	green
MIR499A	green
FAM83C-AS1	green
C20orf173	green
DLGAP4-AS1	green
MYL9	green
TGM2	green
LBP	green
MIR6871	green
LINC01620	green
RBPJL	green
MIR6812	green
UBE2C	green
CD40	green
ZNF663P	green
MKRN7P	green
LOC101927377	green
LOC100131496	green
CSE1L-AS1	green
MIR1302-5	green
MIR645	green
LOC100506175	green
CASS4	green
BMP7-AS1	green
PCK1	green
APCDD1L	green
MIR296	green
TUBB1	green
LOC729296	green
MIR646	green
LOC101928048	green
LAMA5-AS1	green
LAMA5	green
SLCO4A1-AS1	green

LINC00029	green
MIR4326	green
FNDC11	green
LIME1	green
RTEL1	green
TNFRSF6B	green
ZBTB46-AS1	green
MIR941-1	green
MIR647	green
UCKL1-AS1	green
LINC00176	green
LINC01692	green
CYYR1	green
LOC101928269	green
CLDN14	green
D21S2088E	green
LINC00189	green
GRIK1-AS1	green
LOC150051	green
LINC01436	green
KCNJ15	green
DSCAM-AS1	green
DSCAM-IT1	green
PLAC4	green
SNORA91	green
LINC01668	green
ERVH48-1	green
LINC00322	green
LINC00319	green
AATBC	green
LOC105372833	green
DNMT3L	green
KRTAP10-4	green
TSPEAR-AS2	green
COL18A1-AS2	green
COL18A1-AS1	green
LOC100129027	green
FTCD	green
COL6A2	green
HORMAD2-AS1	green
BCRP3	green
SPECC1L-ADORA2A	green
UPB1	green
TMPRSS6	green
PHF21B	green
CELSR1	green

C22orf34	green
LOC101929372	green
MIR3198-1	green
MIR648	green
LINC01634	green
CDC45	green
CLDN5	green
MIR4761	green
MIR185	green
MIR1286	green
LOC284865	green
PI4KAP1	green
LRRC74B	green
LOC102724728	green
CCDC116	green
PRAMENP	green
FBXW4P1	green
C22orf15	green
DERL3	green
SUSD2	green
ADORA2A	green
ADORA2A-AS1	green
POM121L10P	green
CRYBB3	green
IGLL3P	green
LRP5L	green
LINC01422	green
LOC100507657	green
C22orf31	green
ZNRF3-AS1	green
SEC14L4	green
MIR3200	green
SMTN	green
RFPL3S	green
LOC339666	green
MIR6069	green
MIR3909	green
MIR6819	green
CARD10	green
LOC101927051	green
MIR6820	green
CSNK1E	green
LOC105373031	green
APOBEC3D	green
SNORD83B	green
SNORD139	green

SNORD43	green
MGAT3-AS1	green
LOC100130899	green
LOC101927257	green
EP300-AS1	green
LOC105373044	green
MIR33A	green
SHISA8	green
CYP2D7	green
FAM109B	green
OGFRP1	green
SERHL2	green
SERHL	green
RRP7BP	green
A4GALT	green
ATP5L2	green
LOC101927447	green
MIR1249	green
FAM118A	green
LINC01589	green
PRR34	green
PRR34-AS1	green
LOC284933	green
MIR4535	green
IL17REL	green
SCO2	green
CPT1B	green
HYDIN2	green
